# Time-Resolved Metabolomics Reveals Distinct, Cell- and Variety-Dependent Profiles of Prostanoids in Melanoma Cells Exposed to Fruit Extracts from *Cornus mas* and *C. officinalis*: A Pilot Study

**DOI:** 10.3390/ijms27156982

**Published:** 2026-08-03

**Authors:** Łukasz Lewandowski, Małgorzata Krzystek-Korpacka, Daria Mykhailova, Martyna Korbecka, Michał Bryk, Mariusz Fleszar, Paulina Fortuna, Alicja Z. Kucharska, Tomasz Sozański, Jolanta Zalejska-Fiolka, Karolina Mosna, Wioleta Szewczak, Iwona Bednarz-Misa

**Affiliations:** 1 Department of Biochemistry and Immunochemistry, Faculty of Medicine, Wroclaw Medical University, 50-368 Wroclaw, Poland; daria.mykhailova@umw.edu.pl (D.M.); martyna.korbecka@umw.edu.pl (M.K.); m.bryk@student.umw.edu.pl (M.B.); iwona.bednarz-misa@umw.edu.pl (I.B.-M.); 2 Omics Research Center, Wroclaw Medical University, 50-368 Wroclaw, Poland; mariusz.fleszar@umw.edu.pl (M.F.); paulina.fortuna@umw.edu.pl (P.F.); karolina.mosna@umw.edu.pl (K.M.); wioleta.szewczak@umw.edu.pl (W.S.); 3 Department of Fruit, Vegetable and Plant Nutraceutical Technology, Wroclaw University of Environmental and Life Sciences, J. Chełmońskiego 37, 51-630 Wroclaw, Poland; alicja.kucharska@upwr.edu.pl; 4 Department of Preclinical Sciences, Pharmacology and Medical Diagnostics, Wroclaw University of Science and Technology, Wybrzeże Wyspiańskiego 27, 50-370 Wroclaw, Poland; tomasz.sozanski@pwr.edu.pl; 5 Department of Biochemistry, Faculty of Medical Sciences in Zabrze, Medical University of Silesia, 40-055 Katowice, Poland; jzalejskafiolka@sum.edu.pl

**Keywords:** prostaglandins, GAMLSS (hierarchical modeling), iridoid glycosides, anthocyanins, cyclooxygenase-2, bioactive phytochemicals

## Abstract

Chronic inflammation and cyclooxygenase (COX)-2–mediated prostanoid signaling contribute to melanoma progression, yet their modulation by natural products remains poorly defined. We examined the effects of dogwood fruit extracts—Japanese cornel (*Cornus officinalis*) and two European cultivars (‘Uholok’, ‘Yantarnyi’)—on temporal prostanoid dynamics in A375 (primary tumor–derived) and MeWo (metastasis-derived) mela-noma cells. Dynamic changes rather than static levels were modeled using GAMLSS or zero-inflated Gamma models to assess time, cell line, cornel type, and dose effects. Untreated A375 cells showed time-dependent increases in PGE_2_, PGF_2α_, thromboxane B_2_, and 13,14-dihydro-PGE_1_, consistent with inducible COX-2 activation, whereas MeWo cells maintained consistently high prostanoid levels, reflecting constitutive COX-2 expression. *Cornus* extracts modulated these trajectories in a cell- and dose-dependent manner. In A375, low concentrations allowed prostanoid accumulation, while higher doses flattened or reversed these increases; Japanese cornel produced the strongest inhibition, whereas European cultivars showed weaker or cultivar-specific effects. MeWo cells were less responsive, with significant changes emerging only at higher doses. PGD_2_ and 6-keto-PGF_1α_ remained largely unchanged, indicating selective targeting of COX-2–dependent prostanoids. Extracts also reduced accumulation of downstream prostanoids, including 15-deoxy-Δ^12^,^14^-PGJ_2_ and 13,14-dihydro-PGE_1_, particularly in A375 cells. These differences highlight a stronger susceptibility of early-stage melanoma to phytochemical intervention and support cultivar-dependent bioactivity linked to phytochemical composition. In summary, *Cornus* extracts selectively attenuate time-dependent trajectories of tumor-promoting prostanoids—most effectively with Japanese cornel—while sparing homeostatic mediators, warranting further investigation on their potential for melanoma chemoprevention or adjunctive therapy.

## 1. Introduction

Growing health awareness and environmental concerns are driving interest in natural therapies, stimulating research into plant-based medicinal products as alternatives to synthetic drugs [1]. Among these, dogwood is receiving increasing attention due to promising findings, including cytoprotective effects on human skin keratinocytes and fibroblasts [2].

Japanese cornel (*Cornus officinalis* Sieb. et Zucc.) and European cornel (*Cornus mas* L.; cornelian cherry), both from the *Cornus* L. subgenus, are widely distributed plants native to eastern Asia and to southwestern Asia and southern Europe, respectively [3,4]. Their fruits and leaves are used in foods, cosmetics, and folk medicine, including traditional Chinese medicine [5,6]. Their broad applications reflect high contents of flavonoids, alkaloids, terpenoids, and organic acids [4,7,8], associated with antioxidant, anti-inflammatory, antitumor, and immunomodulatory activities [4,9,10,11,12,13]. *Cornus* fruits are particularly rich in iridoids and non-flavonoid glucosides. Although the two species share similar chemical profiles, they differ qualitatively and quantitatively: *C. officinalis* contains numerous iridoids (e.g., morroniside, cornin, sweroside, loganin, 7-O-methylmorroniside, cornuside), whereas *C. mas* contains mainly loganic acid (88–96% of total iridoids), with smaller amounts of cornuside, loganin, and sweroside [3,14,15]. *C. officinalis* also contains moronoside, absent in *C. mas* [16]. Anthocyanin profiles differ as well—*C. mas* accumulates at higher levels, with cyanidin and pelargonidin galactosides predominating depending on cultivar, whereas pelargonidin derivatives predominate in *C. officinalis* [15,16].

The skin, constantly exposed to physical, chemical, and biological stressors, is prone to oxidative stress and inflammation. Persistent reactive oxygen species generated by UV radiation and immune responses damage macromolecules, promoting genetic instability, impaired DNA repair, and carcinogenesis [17]. Malignant melanoma, though comprising a minority of skin cancers, is the most aggressive and lethal, with 303,105 new cases reported globally in 2021. Its incidence continues to rise, especially in high-income regions, driven by UV exposure, fair skin, tanning behaviors, and environmental factors. Mortality, however, has declined due to early detection and advances in immunotherapy and targeted therapies (PD-1 and BRAF/MEK inhibitors). Given melanoma’s high metastatic potential, early diagnosis and preventive sun-protection strategies remain critical [18].

Melanomas overexpress cyclooxygenase-2 (COX-2), which, like COX-1, synthesizes prostaglandin H_2_, the precursor of primary prostanoids: PGD_2_, PGE_2_, PGF_2α_, PGI_2_, and TXA_2_ [19,20,21]. Arachidonic acid also yields leukotrienes such as LTA_4_ via 5-lipoxygenase. These mediators exert diverse and sometimes opposing effects. PGE_2_ is strongly pro-inflammatory and supports tumor development, angiogenesis, immunosuppression, and chemoresistance [19,21,22], whereas PGD_2_ is considered tumor-suppressive; its deficiency accelerates melanoma growth in mice, while PGD_2_/DP-1 activation inhibits it [19,23]. Reflecting their importance in cancer biology, prostanoid-receptor agonists and antagonists are being developed as potential chemopreventive or therapeutic agents, with some already in clinical trials [19].

Given this context, we aimed to profile time-dependent changes in prostanoid concentrations in melanoma A375 and MeWo cells exposed to fruit extracts from three cornels—*C. officinalis* and two *C. mas* cultivars (‘Uholok’ and ‘Yantarnyi’)—using time-resolved metabolomics. This approach captures dynamic metabolic reprogramming not observable in single-time-point analyses [24]. We hypothesized that *Cornus* fruit extracts modulate inflammatory and angiogenic prostanoid responses in melanoma cells in a manner dependent on cornel variety. To shed some light on potential mechanisms, we also examined extract-induced expression changes in prostanoid-related genes: *PTGS2* (encoding COX-2), *mPGES1* (encoding microsomal prostaglandin E synthase), and *HPGD* (encoding 15-hydroxyprostaglandin dehydrogenase 1) using RT-qPCR.

## 2. Results

### 2.1. Baseline Profiles of Lipid Mediators in A375 and MeWo Cells

Concentrations of prostanoids or their stable derivatives in 24−, 48−, and 72-h cultures of untreated A375 and MeWo cells were determined using LC-MS/MS. These included PGE_2_, PGF_2α_, PGD_2_, 5-deoxy-Δ^12^,^14^-PGJ_2_—a PGD_2_ metabolite (further referred to as PGJ_2_), 13,14-dihydro-PGE_1_—a PGE_1_ metabolite (further referred to as PGE_1_), 6-ketoPGF_1α_—a stable metabolite of PGI_2_ (further referred to as PGF_1α_), and TXB_2_—a stable metabolite of TXA_2_. Concentrations of LTB_4_—a stable LTA_4_ metabolite—were determined as well.

**Figure 1:**
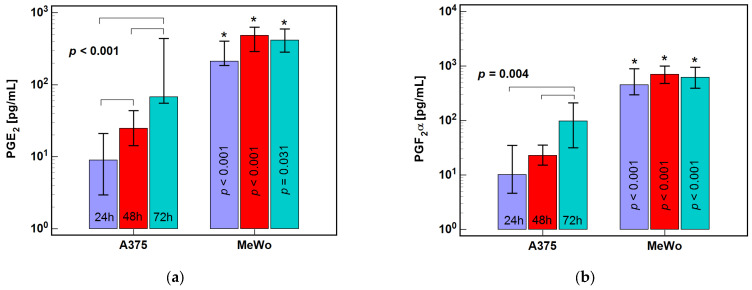

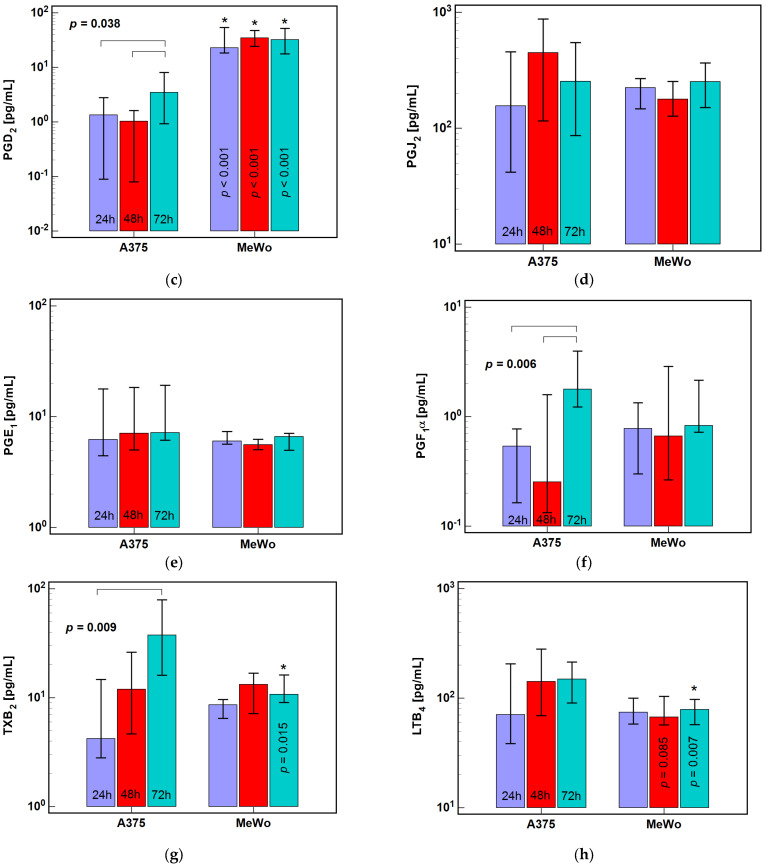
Baseline secretion of lipid mediators by A375 and MeWo melanoma cells after 24 h (violet bars), 48 h (red bars), and 72 h (green bars): (**a**) PGE_2_; (**b**) PGF_2α_; (**c**) PGD_2_; (**d**) PGJ_2_; (**e**) PGE_1_; (**f**) PGF_1α_; (**g**) TXB_2_ and (**h**) LTB_4_. Data presented as median concentrations with 95% confidence intervals (bars with whiskers). The effect of time (24 h vs. 48 h vs. 72 h) within each cell line was analyzed using the Kruskal–Wallis *H* test with the Conover post hoc test, and significant (*p* < 0.05) between-group differences are indicated by connectors. Effect of cell type (MeWo vs. A375) was analyzed for each time-point using the Mann–Whitney *U* test with significant probabilities indicated by asterisks and *p*-values shown within bars.

Baseline concentrations of PGE_2_, PGF_2α_, PGD_2_, PGF_1α_, and TXB_2_ in A375 cells increased with culture duration, while that of PGJ_2_, PGE_1_, and LTB_4_ was not significantly altered. MeWo, in turn, displayed a stable synthesis of prostanoids and LTB_4_ over time (Figure 1).

Regardless of culture duration, MeWo cells synthesized significantly more PGE_2_, PGF_2α_, and PGD_2_ than A375 cells. Instead, they produced less TXB_2_ and LTB_4_ after 72 h than A375 (Figure 1). Median metabolite concentrations in untreated cells depending on their type and culture duration are provided in the Supplementary Materials in Table S1.

### 2.2. Dynamics of Lipid Mediators upon Exposure to Cornel Extract (Time and Its Hierarchical Modulators)

Cells were treated with extracts from Japanese (*C. officinalis*) cornel and ‘Uholok’ and ‘Yantarnyi’ cultivars of European (*C. mas*) cornel. The effects of exposure time, extract type and concentration, and cell line type, as well as their interactions, were analyzed to model the dynamics in the synthesis of lipid mediators PGE_2_, PGF_2α,_ PGD_2_, PGJ_2_, PGE_1_, PGF_1α_, TXB_2_, and LTB_4_. Each developed model is parsimonious, that is, it consists only of effects/interactions which substantially added to the model’s fit to the data. For clarity purposes, non-meaningful effects/interactions were excluded. The goodness-of-fit is shown in the ‘Norma Q-Q plots’ in Tables S2–S9 of the Supplementary Materials. Full model architectures, diagnostic plots, and estimated simple effects are also provided in Tables S2–S9 as well as in the ‘readme’ file. Reported simple effects correspond to prespecified concentrations (27, 272, 518, and 763 µg/mL) and to an exposure time of 48 h.

#### 2.2.1. Model for PGE_2_

**Figure 2:**
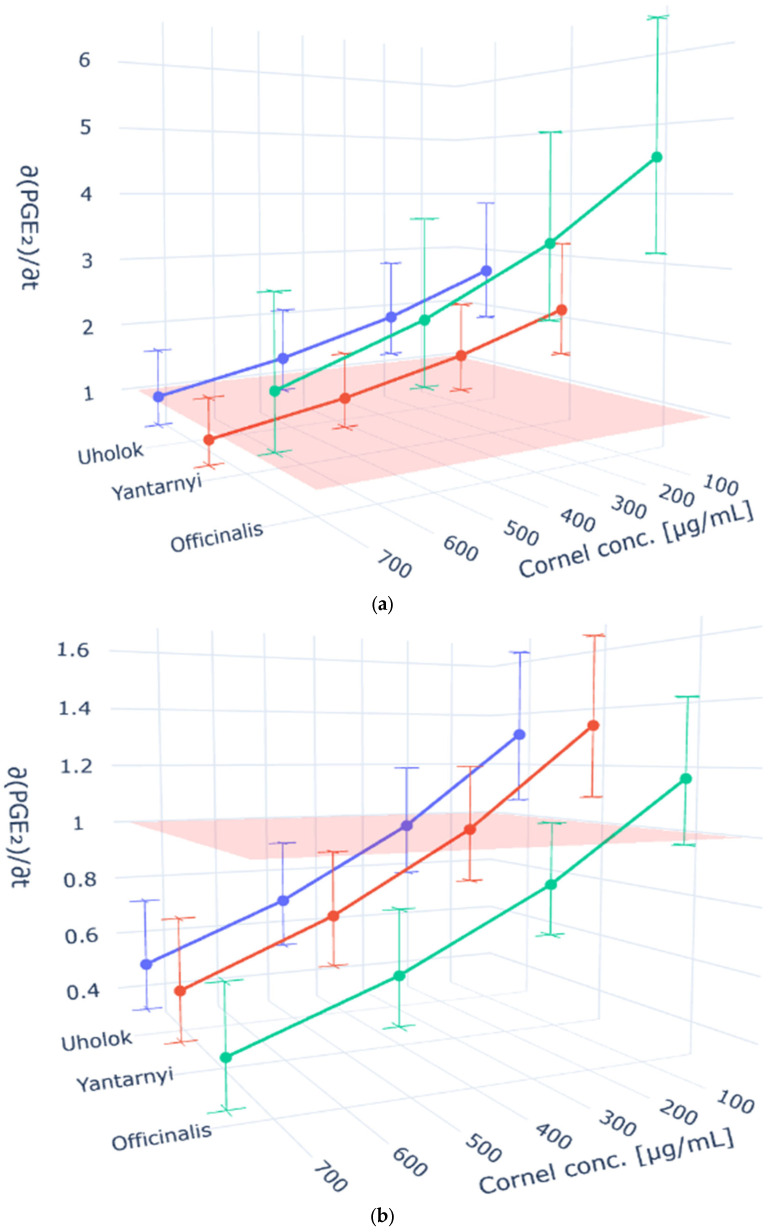
The effect of the type and concentration of cornel extract on PGE_2_ synthesis during exposure in: (**a**) A375 cells and (**b**) MeWo cells. The relationship between PGE_2_ concentration and exposure time is presented as the slopes of PGE_2_ quantity over time while keeping other variables in the model constant, with 95% confidence intervals (whiskers). Officinalis, Japanese cornel (*C. officinalis*); Uholok, European cornel (*C. mas*) ‘Uholok’ cultivar; Yantarnyi, European cornel (*C. mas*) ‘Yantarnyi’ cultivar; conc. concentration.

Temporal trajectories of PGE_2_ (change in prostaglandin synthesis over time), taking into account the type and concentration of cornel extract, are shown for each cell line in Figure 2a,b (interactive 3D visualizations are available in Supplementary Materials: model1_A375 and MeWo).

A strong interaction between time and extract concentration (‘time × extract’, *β_i_* = −0.300, *p* < 0.001; Table 1) indicates that the temporal trajectory of PGE_2_ is dose-dependent.

In A375 cells, PGE_2_ concentrations rose sharply over time at low extract dose, with a slope of *β* = 1.509 at 26.6 µg/mL. However, this increase progressively flattened as extract dose increased, reaching near-neutral values at 763 µg/mL (*β* = 0.610; Table S2).

In MeWo cells, the pattern was strikingly different: at low extract concentrations there was little to no temporal change (*β* = 0.153), but higher doses induced a clear decline over time, with *β* = −0.746 at 763 µg/mL (Table S2).

These differences were also reflected in baseline slopes at 272 µg/mL, where A375 treated with Japanese cornel extract showed a strong positive slope (*β* = 1.209), while MeWo exhibited no significant change (*β* = −0.146; Table S2). The negative ‘time × cellsM’ interaction (*β_i_* = −1.356, *p* < 0.001; Table 1) confirms that MeWo has a consistently weaker, and often opposite, temporal trajectory compared to A375.

Extract type also modulated temporal changes. In A375, the ‘Uholok’ and ‘Yantarnyi’ cultivars of European cornel produced lower baseline slopes than Japanese cornel extract (‘time × varietyU’: *β*_i_ = −0.537, *p* = 0.038; ‘time × varietyY’: *β*_i_ = −0.743, *p* = 0.005; Table 1). At baseline concentration, ‘Uholok’ showed *β* = 0.672, while ‘Yantarnyi’ showed *β* = 0.466, compared with *β* = 1.209 for Japanese cornel extract (Table S2). However, there was no significant three-way interaction between time, concentration, and type, indicating that type-related differences primarily reflect baseline trajectory shifts rather than systematic modulation of the dose–response pattern.

In MeWo, the effects of extract type were also present but more modest. At baseline concentration, temporal slopes were slightly negative but non-significant for ‘Uholok’ (*β* = −0.025) and ‘Yantarnyi’ (*β* = −0.007). Positive three-way interactions (‘time × cellsM × varietyU’: *β*_i_ = 0.659, *p* = 0.025; ‘time × cellsM × varietyY’: *β* = 0.883, *p* = 0.003; Table 1) show that type-related differences between A375 and MeWo are context-specific, but again, they do not involve systematic dose modulation.

Overall, these results demonstrate that extract concentration is the primary driver of temporal PGE_2_ dynamics. A375 responds to higher concentrations with a progressive flattening of its initially steep upward trajectory, while MeWo shifts from minimal change at low to a pronounced decline at high extract doses. Differences between extract types at baseline were statistically significant but modest and largely limited to shifts in initial trajectories rather than alterations in the dose–response relationship.

#### 2.2.2. Model for PGF_2α_

**Figure 3:**
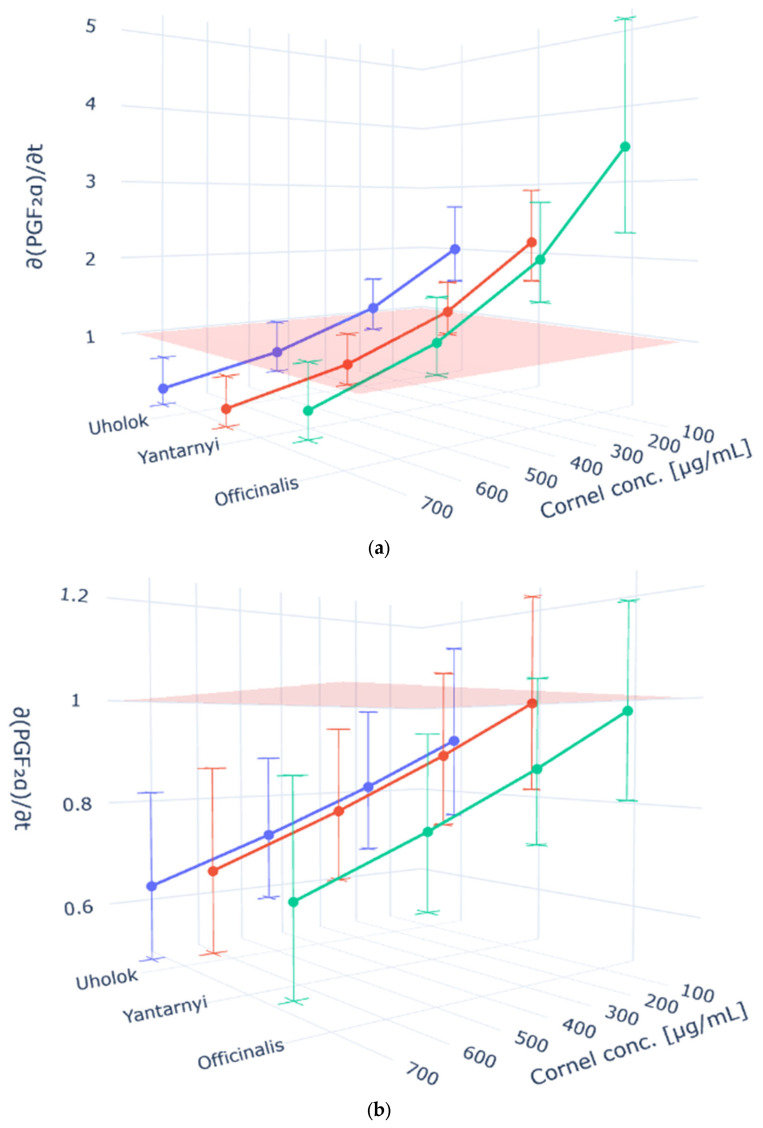
The effect of the type and concentration of cornel extract on PGF_2α_ synthesis during exposure in: (**a**) A375 cells and (**b**) MeWo cells. The relationship between PGF_2α_ concentration and exposure time is presented as the slopes of PGF_2α_ quantity over time while keeping other variables in the model constant, with 95% confidence intervals (whiskers). Officinalis, Japanese cornel (*C. officinalis*); Uholok, European cornel (*C. mas*) ‘Uholok’ cultivar; Yantarnyi, European cornel (*C. mas*) ‘Yantarnyi’ cultivar; conc. concentration.

Temporal trajectories of PGF_2α_, taking into account the type and concentration of cornel extract, are shown for each cell line in Figure 3a,b (interactive 3D visualizations are available in Supplementary Materials: model2_A375 and MeWo).

Temporal changes in PGF_2_α concentrations differed sharply between the two melanoma cell lines and were strongly modulated by extract concentration (‘time × extract’, *β_i_* = −0.527, *p* < 0.001; Table 2).

At the lowest extract dose of 26.6 µg/mL, PGF_2α_ concentrations in A375 cells strongly increased over time with a slope of *β* = 1.239 (Table S3). As extract dose rose, this upward trend progressively weakened, reaching a nearly neutral slope at 518 µg/mL (*β* = 0.186; Table S3) and approaching reversal at the highest extract concentration. For Japanese cornel extract, the high-dose slope trended negative but was not statistically significant (763 µg/mL: *β* = −0.341; Table S3). By contrast, ‘Uholok’ and ‘Yantarnyi’ cultivars of European cornel showed significant declines at the same high dose with *β* = −0.883 for ‘Uholok’ and *β* = −0.810 for ‘Yantarnyi’ (Table S3).

At baseline concentration (272 µg/mL), A375 cells still exhibited a positive slope in the case of Japanese cornel extract (*β* = 0.713; Table S3), which was substantially reduced for both cultivars of European cornel: *β* = 0.171 for ‘Uholok’ and *β* = 0.244 for ‘Yantarnyi’ (Table S3).

Temporal changes in MeWo cells were consistently smaller in magnitude, as indicated by the strong negative ‘time × cellsM’ term (*β_i_* = −0.849, *p* < 0.001; Table 2). At low extract concentrations, MeWo showed no significant temporal change (*β* = −0.021 for 26.6 µg/mL; Table S3). However, with increasing extract dose, slopes became progressively negative, indicating a dose-dependent decline with *β* = −0.251 at 518 µg/mL and *β* = −0.366 at 763 µg/mL (Table S3). This weaker, as compared to A375 cells, dose-related effect is reflected in the positive ‘time × cellsM × extract’ interaction (*β_i_* = 0.412, *p* = 0.001; Table 2).

At baseline extract concentration, MeWo exhibited no significant temporal effect in the case of Japanese cornel (*β* = −0.136), a small but significant decline for ‘Uholok’ (*β* = −0.197), and a non-significant decline for ‘Yantarnyi’ (β = −0.114) (Table S3). Positive interactions for ‘time × cellsM × varietyU’ (*β_i_* = 0.481, *p* = 0.036; Table 2) and ‘time × cellsM × varietyY’ (*β_i_* = 0.491, *p* = 0.022; Table 2) indicate context-dependent differences between the two cell lines.

Dose contrasts further highlight the stronger sensitivity of A375 to concentration changes: increasing the extract dose by 245.58 units changed the time slope by β = −0.527 in A375 cells against *β* = −0.115 in MeWo (Table S3). No ‘time × type × concentration’ term was included in the model, indicating that there is no statistical evidence for a systematic three-way modulation of concentration effects by cornel variety.

Overall, these results demonstrate that extract concentration is the primary driver of PGF_2_α temporal dynamics. A375 shows a steep temporal increase at low extract doses, which is progressively dampened and ultimately reversed at high doses, especially when exposed to ‘Uholok’ and ‘Yantarnyi’ cultivars of European cornel. MeWo exhibits weaker, consistently negative concentration effects, with declines that never reach the magnitude observed in A375 cells.

#### 2.2.3. Model for PGD_2_

Temporal changes in PGD_2_ concentrations differed between melanoma cell lines and cornel varieties (Table 3). However, extract concentration was not included in this model, indicating that PGD_2_ dynamics are independent of extract dose. Estimated simple effects, full model architectures, and diagnostic plots are provided in Table S4.

#### 2.2.4. Model for 15-Deoxy-Δ^12^,^14^-PGJ_2_ (PGJ_2_)

**Figure 4:**
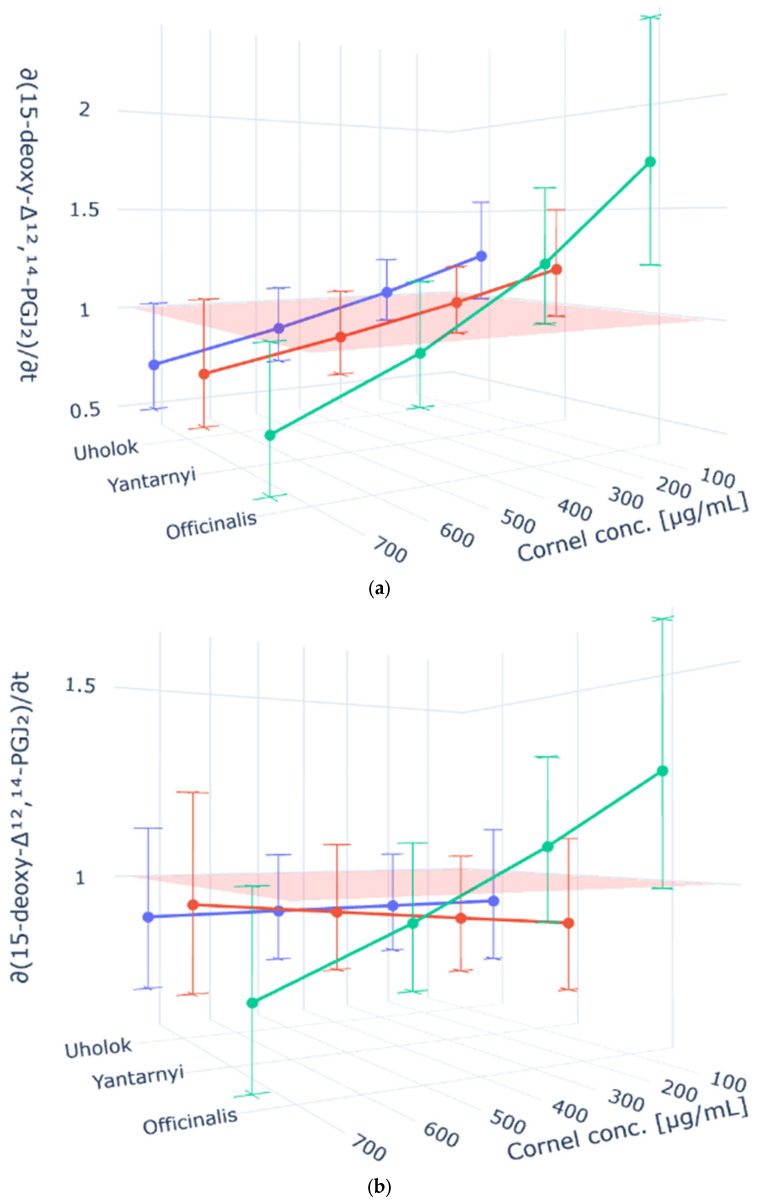
The effect of the type and concentration of cornel extract on 15-deoxy-Δ^12^,^14^-PGJ_2_ synthesis during exposure in: (**a**) A375 cells and (**b**) MeWo cells. The relationship between 15-deoxy-Δ^12,14^-PGJ_2_ concentration and exposure time is presented as the slopes of 15-deoxy-Δ^12^,^14^-PGJ_2_ quantity over time while keeping other variables in the model constant, with 95% confidence intervals (whiskers). Officinalis, Japanese cornel (*C. officinalis*); Uholok, European cornel (*C. mas*) ‘Uholok’ cultivar; Yantarnyi, European cornel (*C. mas*) ‘Yantarnyi’ cultivar; conc. concentration.

Changes in PGJ_2_ synthesis over time, taking into account the type and concentration of cornel extract, are shown for each cell line in Figure 4a,b (interactive 3D visualizations are available in Supplementary Materials: model4_A375 and MeWo).

The effect of extract concentration on temporal changes in 15-deoxy-Δ^12^,^14^-PGJ_2_ concentrations was cell line– and cornel variety–specific, as indicated by significant ‘time × extract’ and higher-order interactions (Table 4).

In A375, increasing concentration of Japanese cornel extract reduced the temporal slope (*β_i_* for ‘time × extract’ = −0.316, *p* = 0.002; Table 4). Simple effects corroborate this dose–response: at low concentration the slope is positive (*β* = 0.543), while at the highest concentration it approaches reversal (*β* = −0.407; Table S5).

In MeWo, temporal changes were weaker. Although ‘time × extract’ was negative overall, the positive ‘time × cellsM × extract’ term (*β_i_* = 0.164, *p* = 0.040) indicates attenuation of the inhibitory effect in MeWo relative to A375. Dose contrasts in MeWo showed a small but detectable reduction (*β* = −0.153), whereas slopes at fixed doses had confidence intervals overlapping 0 (Table S5).

Across cornel varieties, Japanese cornel extract showed the strongest inhibition. The ‘Uholok’ cultivar of European cornel exhibited a borderline modification of the dose effect (‘time × varietyU × extract’: *β_i_* = 0.152, *p* = 0.065; Table 4), and simple effects for A375 suggest a weak suppression with confidence interval narrowly excluding 0 (*β* = −0.164; Table S5). The ‘Yantarnyi’ cultivar of European cornel displayed a significant positive modification relative to Japanese cornel (‘time × varietyY × extract’: *β_i_* = 0.185, *p* = 0.031; Table 4), consistent with attenuation of the inhibitory dose effect (simple effects generally with confidence intervals overlapping zero; Table S5).

#### 2.2.5. Model for 13,14-Dihydro-PGE_1_

The effect of extract concentration on temporal changes in 13,14-dihydro-PGE_1_ concentrations was cell-line-specific, as indicated by significance of the ‘time × cellsM × extract’ interaction (Table 5).

**Figure 5:**
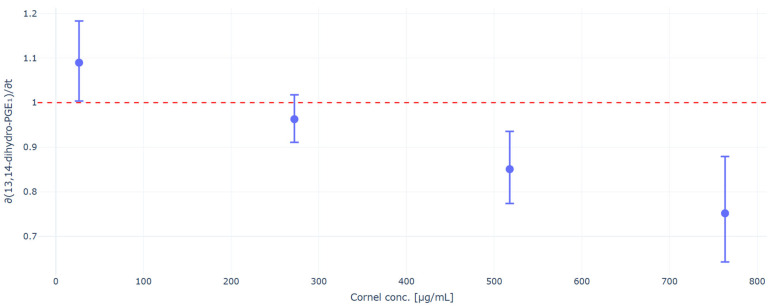
The effect of cornel extract concentration on 13,14-dihydro-PGE_1_ synthesis during exposure in A375 cells. Relationship between 13,14-dihydro-PGE_1_ concentration and exposure time is presented as slopes of 13,14-dihydro-PGE_1_ quantity over time while keeping other variables in the model constant, with 95% confidence intervals (whiskers). Conc. concentration.

In A375 cells, increasing extract concentration progressively reduced the temporal slope (Figure 5) (interactive 3D visualization is available in Supplementary Materials: model5_A375).

At the lowest concentration (26.6 µg/mL), the slope was slightly positive and significant (*β* = 0.086; Table S6), reflecting an intrinsic increase in 13,14-dihydro-PGE_1_ over time. At higher concentrations, however, this trend flattened and ultimately became negative, with *β* = −0.162 at 518 µg/mL and *β* = −0.285 at 763 µg/mL (Table S6).

The overall dose effect was strongly negative (*β_i_* = −0.124, *p* = 0.001; Table 5), indicating a clear inhibitory action of the extract on temporal 13,14-dihydro-PGE_1_ dynamics in A375.

In MeWo cells, temporal slopes remained close to zero across all concentrations, with confidence intervals (CI) consistently overlapping zero (e.g., baseline *β* = 0.0002, 95% CI = −0.024 to 0.024; Table S6). Although both ‘time × extract’ and ‘time × cellsM × extract’ were individually significant, they had opposite signs and thus canceled each other when combined, yielding no net effect of concentration on MeWo trajectories (Table 5).

Overall, these results show that extract concentration strongly suppresses the temporal increase in 13,14-dihydro-PGE_1_ in A375 cells but does not affect MeWo, highlighting a cell-line-specific response to treatment.

#### 2.2.6. Model for 6-Keto-PGF_1α_

There was no effect of extract concentration on the temporal slope for 6-keto-PGF_1α_, as no ‘time × extract’ term was included in the model. Instead, temporal changes depended on the type of cell line and, to a lesser extent, on the corn variety (Table 6). Estimated simple effects, full model architectures, and diagnostic plots are provided in Table S7.

#### 2.2.7. Model for TXB_2_

**Figure 6:**
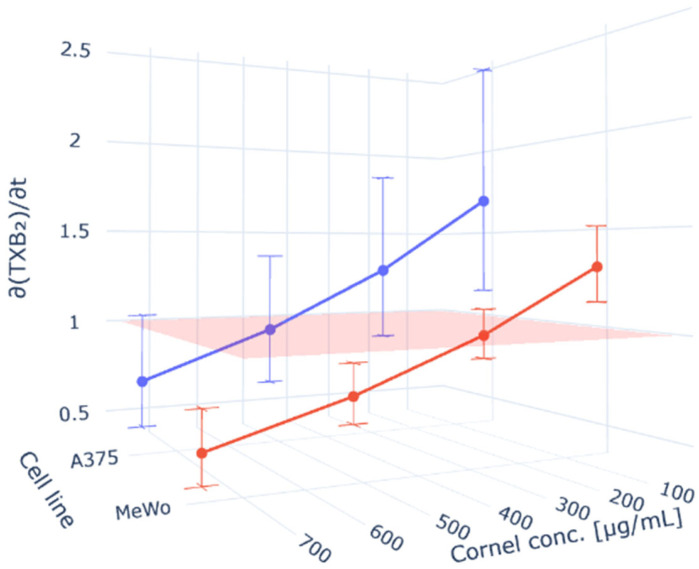
The effect of the extract concentration of Japanese cornel (*C. officinalis*) on TXB_2_ synthesis during exposure in A375 and MeWo cells. The relationship between TXB_2_ concentration and exposure time is presented as the slopes of TXB_2_ quantity over time while keeping other variables in the model constant, with 95% confidence intervals (whiskers). Conc. concentration.

Temporal changes in TXB_2_ were primarily shaped by extract concentration, as indicated by a strong negative ‘time × extract’ interaction (*β_i_* = −0.280, *p* < 0.001). Rising concentrations progressively dampened temporal changes, with higher extract doses leading to markedly lower slopes compared to baseline (Figure 6; Table 7) (interactive 3D visualization is available in Supplementary Materials: model7).

In A375 cells, temporal changes were strongly positive at low extract concentrations but diminished when they increased. At the lowest concentration of 26.6 µg/mL, the slope was strongly positive (*β* = 0.526; Table S8), indicating a clear temporal increase. At baseline extract concentration of 272 µg/mL, the slope was smaller and not statistically significant (*β* = 0.246; Table S8). With higher doses, slopes approached neutral or negative values, that is, *β* = −0.034 at 518 µg/mL and *β* = −0.314 at 763 µg/mL (Table S8). This pattern suggests a non-linear dose–response, with a sharp increase at low extract doses that is flattened and eventually reversed at high extract doses.

At baseline concentration, A375 showed clear differences between cornel varieties. Compared to Japanese cornel, extract from the ‘Uholok’ cultivar of European cornel produced a significantly higher positive slope (*β* = 0.651), while the one produced by extract from the ‘Yantarnyi ‘ cultivar was even more positive (*β* = 0.798; Table S8). However, their effects on the concentration-dependent dynamics were weaker and less consistent. This is reflected in the positive ‘time × varietyU × extract’ interaction for ‘Uholok’ (*β_i_* = 0.226, *p* = 0.013; Table 7) and the borderline ‘time × varietyY × extract’ interaction for ‘Yantarnyi’ (*β_i_* = 0.162, *p* = 0.062; Table 7), indicating that these variants somewhat reduced the dose-dependent suppression seen with the medicinal extract.

Temporal patterns in MeWo cells differed markedly. At baseline, MeWo exhibited little to no intrinsic temporal change for Japanese cornel extract (*β* = −0.011; Table S8). With increasing extract concentration, the slopes became progressively negative, showing a clear dose-dependent decline with *β* = −0.291 at 518 µg/mL and *β* = −0.571 at 763 µg/mL (Table S8). Even at the lowest extract dose (26.6 µg/mL), a modest increase was detectable (*β* = 0.269; Table S8), illustrating a gradual shift from slight early increases to sharp declines at high concentrations.

Differences between A375 and MeWo were not statistically significant at baseline (‘time × cellsM’, *β_i_* = −0.257, *p* = 0.167; Table 7), but the variety-specific interactions approached significance, with *β_i_* = −0.445 (*p* = 0.056) for the ‘time × cellsM × varietyU’ effect and *β_i_* = −0.552 (*p* = 0.121; Table 7) for ‘time × cellsM × varietyY’ effect. These terms suggest that the stronger baseline temporal increases seen in A375 treated with ‘Uholok’ and ‘Yantarnyi’ cultivars of European cornel are less pronounced in MeWo melanoma cells.

Overall, Japanese cornel extract was the most consistent in suppressing temporal TXB_2_ changes in a clear, dose-dependent manner. In contrast, ‘Uholok’ and ‘Yantarnyi’ cultivars of European cornel displayed weaker modulation of the temporal trajectory, with ‘Yantarnyi’ showing only borderline significance. In A375, this resulted in a steep positive slope at low doses that was neutralized or reversed at high extract doses. In MeWo, the direction of temporal change remained consistent across extract concentrations, transitioning smoothly from modest increases at low doses to sharp declines at high doses.

#### 2.2.8. Model for LTB_4_

Temporal dynamics of LTB_4_ showed clear differences between cell lines and corn varieties, but extract concentration had no significant global effect on these trajectories (‘time × extract’, *p* = 0.129; Table 8). The only signal related to concentration was a very small and borderline interaction for the ‘Uholok’ cultivar (‘time × varietyU × extract’: *β*_i_ = 0.125, *p* = 0.047; Table 8).

However, simple effects for ‘Uholok’ at different concentrations had confidence interval overlapping zero (e.g., *β* = 0.056; Table S9), indicating that this finding is weak and may not be biologically meaningful.

### 2.3. Baseline Expression Profiles of PTGS2, mPGES1, and HPGD in A375 and MeWo Cells

**Figure 7:**
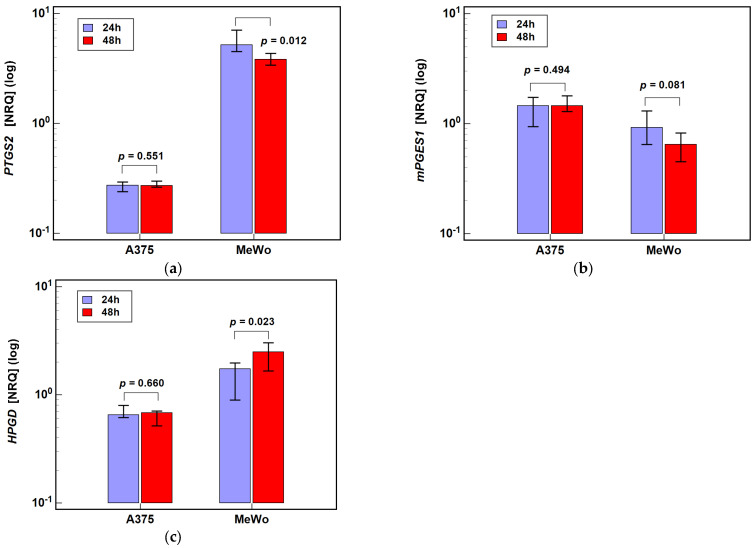
Baseline expression profiles of *PTGS2*, *mPGES1*, and *HPGD* in A375 and MeWo melanoma cells after 24 and 48 h: (**a**) *PTGS2*; (**b**) *mPGES1*; (**c**) *HPGD*. Data presented as mean concentrations with 95% confidence intervals (bars with whiskers). The effect of time was analyzed using a *t*-test for independent samples with Welch correction, with *p*-values for between-group differences indicated by connectors. NRQ, normalized relative quantities.

Baseline expression of *PTGS2*, *mPGES1*, and *HPGD* is stable over time in A375 cells, while in MeWo, *PTGS2* and *mPGES1* expression declines, significantly so for *PTGS2*, and that of *HPGD* increases in 48-h cultures as compared to 24-h cultures (Figure 7).

MeWo cells expressed significantly more *PTGS2* than A375: by 20.6-fold (*p* < 0.0001) in 24-h and by 14.1-fold (*p* < 0.0001) in 48-h cultures. Likewise, *HPGD* expression was significantly higher: by 2.1-fold (*p* < 0.001) and 3.5-fold (*p* < 0.0001) in 24-h and 48-h cultures, respectively. In turn, *mPGES1* expression was lower in MeWo, significantly so in 48-h (by 2.6-fold, *p* < 0.001) cultures.

### 2.4. Impact of Cornel Extracts on Expression Profiles of PTGS2, mPGES1, and HPGD in A375 and MeWo Cells

**Figure 8:**
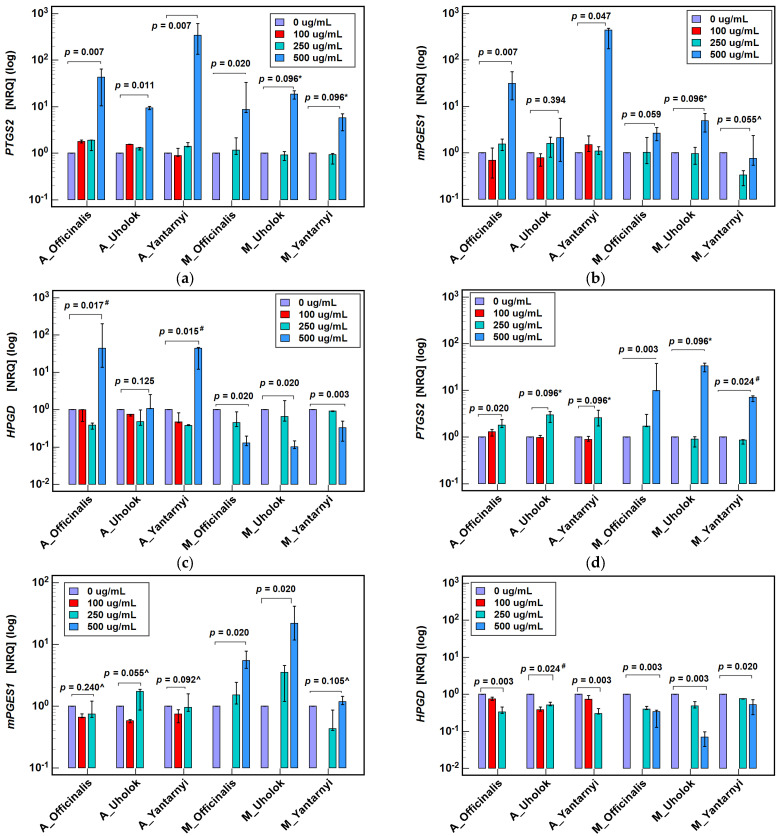
Impact of cornel extracts on expression profiles of *PTGS2*, *mPGES1*, and *HPGD* in A375 and MeWo cells: (**a**) *PTGS2* after 24-h exposure; (**b**) *mPGES1* after 24-h exposure; (**c**) *HPGD* after 24-h exposure; (**d**) *PTGS2* after 48-h exposure; (**e**) *mPGES1* after 48-h exposure; (**f**) *HPGD* after 48-h exposure. Data presented as median concentrations with interquartile range (bars with whiskers). If not otherwise stated, concentration effect was analyzed using the Jonckheere-Terpstra trend test. ^, data analyzed using Kruskal–Wallis *H* test with Conover post hoc test; #, each group differs significantly from others in Kruskal–Wallis *H* test; *, *p* = 0.055 in Kruskal–Wallis *H* test. NRQ, normalized relative quantities; A_, expression in A375 cells; M_, expression in MeWo cells.

Japanese cornel extract dose-dependently upregulated *PTGS2* expression in both cell lines and exposure times. The *mPGES1* expression also displayed an increasing trend, although low extract doses rather reduced *mPGES1* in A375 cells. A375 cells responded to 24-h treatment with ‘Uholok’ and ‘Yantarnyi’ extracts with a rise in *PTGS2,* while 48-h exposure tended to slightly reduce gene expression at lower and increase at higher concentrations, similarly to MeWo response to 24− and 48-h exposure (Figure 8). A tendency towards a similar dichotomous pattern could be observed in the case of *mPGES1*, except for a dose-dependent increase in gene expression following 48-h treatment of MeWo cells with ‘Uholok’ cultivar (Figure 8).

Both cell lines responded to cornel extracts, regardless of their type and exposure length, with a dose-dependent downregulation of *HPGD*, except for an increase at 500 μg/mL observed in A375 cells (Figure 8).

Comparisons of gene expression at given extract type and concentrations showed significant differences with respect to exposure length. Longer exposure of A375 to the ‘Uholok’ cultivar of European cornel resulted in lower *PTGS2* (by 1.5-fold, *p* = 0.001) and *HPGD* expression (by 1.8-fold, *p* = 0.006) at 100 μg/mL concentration and in 2.2-fold higher *PTGS2* expression (*p* = 0.022) at 250 μg/mL concentration. In addition, longer exposure to the ‘Yantarnyi’ cultivar at 100 μg/mL resulted in 2.2-fold lower *mPGES1* expression (*p* = 0.082) and to Japanese cornel at the same concentration to 1.4-fold lower *PTGS2* expression (*p* = 0.052).

In turn, longer exposure of MeWo cells to high concentrations (500 μg/mL) of ‘Uholok’ led to 1.7-fold higher *PTGS2* (*p* = 0.072) and 4.9-fold higher *mPGES1* expression (*p* = 0.057), and of Japanese cornel to 2.2-fold higher *mPGES1* expression (*p* = 0.089).

## 3. Discussion

To our knowledge, this is the first study investigating cornel’s effect on prostanoid and leukotriene profiles in melanoma cells, as well as the first to model metabolite dynamics alongside higher-order interactions between potential effectors, providing a more realistic representation of biological systems. Importantly, the present findings should be interpreted across three complementary analytical layers. Section 2.1 reports absolute concentrations of prostanoids measured at discrete time points, Section 2.2 describes their temporal dynamics using model-based estimates of time-dependent change, and Section 2.3 and Section 2.4 provide baseline expression profiles of selected genes involved in the metabolism of prostanoids. These layers capture related but distinct biological quantities and should not be expected to mirror one another directly. Notably, the modeling framework in 2.2 (GAMLSS) controlled dispersion of the measurements, which could stem from laboratory-associated artifacts in the data.

Dynamic modeling using hierarchical frameworks offers a fundamentally different perspective compared to conventional fixed-timepoint analyses. Whereas the latter focus on static measurements of metabolite abundance (quantities that are tightly coupled to experimental conditions), dynamic models quantify the rate of change (i.e., the temporal partial derivative) of metabolite production. In the present study, time was treated as a continuous covariate (24, 48, and 72 h), such that GAMLSS estimates should be interpreted as smoothed, model-based summaries of overall kinetics rather than as direct encodings of pairwise differences between individual measurements. This distinction is more than methodological nuance: it allows the results to reflect intrinsic cellular behaviors rather than laboratory artifacts, since the kinetics of prostanoid release are a biological property of melanoma cells, while absolute concentrations are context-dependent snapshots.

Skin is continuously exposed to environmental stressors that promote oxidative stress and chronic inflammation—conditions known to facilitate tumor development through induction of pro-carcinogenic mediators such as COX-2 and its prostanoid products. While the inflammatory component of skin carcinogenesis has been extensively studied in non-melanoma skin cancers, its role in melanoma remains less well understood [17]. Nevertheless, overexpression of COX-2/*PTGS2* has been consistently documented in melanoma tissues [25,26,27,28,29]. Although Goulet et al. [26] observed COX-2 solely in melanocytes from lymph nodes and distant metastases, subsequent analyses involving larger cohorts demonstrated robust COX-2 protein expression in primary tumors, localized mainly to malignant melanocytes [27].

COX-2/*PTGS2* expression has also been confirmed in melanoma cell lines [25,26,30,31], though its basal levels vary and reports regarding individual lines are not coherent. Unstimulated A375 cells exhibit relatively low COX-2 mRNA and protein levels compared to MeWo cells, whereas COX-1 expression remains high [26,30,31]. Consistent with these findings, *PTGS2* mRNA levels were higher in MeWo than A375 cells, as were the levels of *HPGD*, encoding the PGE_2_-degrading enzyme. Still, the expression of *mPGES1*—encoding the other enzyme involved in PGE_2_ synthesis—was lower. All major prostanoids examined herein were synthesized in both A375 and MeWo cultures, but the intracellular concentrations of PGE_2_, PGF_2α_, and PGD_2_ were significantly higher in MeWo cells, derived from lymph node metastasis. The comparatively weaker prostaglandin synthesis in A375 agrees with its primary tumor origin and the predominance of COX-1 activity at baseline [26,31]. The time-dependent increase in prostaglandin production observed in A375 may be compatible with COX-2-related signaling, consistent with previous reports linking its expression to mitotic rate of melanomas [28,30]. Notably, this interpretation refers to baseline temporal trends and does not imply a direct or proportional coupling between COX-2 transcriptional activity and short-term prostanoid flux. In contrast, prostaglandin concentrations in fibroblast-like MeWo remained stably elevated over time, a profile resembling COX-2 expression kinetics described in fibroblasts. Between-line differences in BRAF^V600E^ mutation status [32] could not contribute to distinct profiles of prostanoids, as neither BRAF^V600E^ nor NRAS ^Q61^ mutations have been associated with altered enzyme expression [31]. Overall, this dichotomy may support a scenario in which primary melanoma cells rely on inducible COX-2 signaling during proliferation, and metastatic cells may have already established a steady-state prostanoid environment supporting survival, motility, and immune evasion.

Baseline gene expression analyses presented in Section 2.3 complement these observations by characterizing the transcriptional potential of enzymes involved in prostanoid turnover, including *PTGS2*, *mPGES-1*, and *HPGD*. However, transcriptional profiles primarily inform on molecular capacity rather than on short-term metabolite flux. Accordingly, concurrent upregulation of COX-2–related genes and higher absolute prostanoid concentrations at fixed time points do not necessitate a proportional increase in the temporal slopes estimated by GAMLSS. Dissociations between expression patterns, absolute concentrations, and kinetic parameters are therefore biologically plausible and reflect regulation at post-transcriptional, enzymatic, and metabolic levels. Such dissociations are therefore expected when temporal dynamics are modeled as continuous processes rather than inferred from discrete measurements.

COX-2/*PTGS2* expression has been ascribed prognostic significance in melanoma [27,28,30,33]. High COX-2 levels (≥10%) have been associated with significantly reduced progression-free survival [31]. Given its frequent expression in melanoma [34] and the well-established pro-tumorigenic role of its metabolites [21,34,35], COX-2 represents an attractive therapeutic target. Knockout models have demonstrated reduced skin carcinogenesis in COX-1- or COX-2-deficient mice [36]. In vitro and in vivo studies have shown that genetic or pharmacological inhibition of COX-2 suppresses melanoma invasion, partly through downregulation of MCP-1 synthesis [37], and that deletion of the microsomal prostaglandin E2 synthaseabolishes tumor-associated immunosuppression and reduces tumor growth [38]. Moreover, the selective COX-2 inhibitor celecoxib downregulates PD-L1 expression in melanoma cells [39]—a co-stimulator responsible for T-cell anergy and apoptosis and ensuing cancer cell evasion [40]—thus potentially enhancing immune recognition. Combined therapy using nonsteroidal anti-inflammatory drugs (NSAIDs) and immune checkpoint inhibitors has further improved tumor reduction in murine melanoma models [41].

However, the long-term use of NSAIDs remains limited by their systemic toxicity, particularly cardiotoxicity [42]. This limitation underscores the need for safer alternatives, such as bioactive natural products with comparable efficacy but reduced adverse effects [43]. Melanoma is characterized by remarkable genetic heterogeneity and cellular plasticity, leading to high mutation rates and the development of drug resistance—a major challenge in current therapies [44]. Phytochemicals can sensitize cancer cells to chemotherapy and help overcome resistance by modulating multiple signaling cascades while exhibiting low intrinsic toxicity [45]. A key advantage of natural metabolites lies in their polypharmacological profile, enabling simultaneous modulation of multiple signaling pathways implicated in cancer progression [46]. This multi-target activity enhances therapeutic efficacy, reduces the likelihood of resistance development, and may lower the risk of adverse interactions associated with polypharmacy [47]. Notably, the isoflavone genistein effectively attenuated mitogenic PGE_2_/EP_3_/IL-8 signaling [48], whereas selective inhibition of COX-2 with NS-398 failed to suppress melanoma cell proliferation [37]. Several other natural compounds targeting the COX-2 pathway have shown anti-melanoma potential, e.g., grape-seed proanthocyanidins reduce melanoma cell migration by interfering with PGE_2_/EP_2_/EP_4_-activated β-catenin signaling and suppressing MMP-2/9 expression [49].

Cornel extracts have demonstrated anti-inflammatory and immunomodulatory effects primarily in metabolic disease models [3,12,50,51,52,53,54,55,56,57], yet emerging evidence suggests potential anticancer activity. Accordingly, juice and fruit extracts from European cornel decrease the viability of colon, breast, liver, and melanoma cancer cells, including A375 and MeWo cells [5,58]. Cornel-derived phytochemicals, including iridoids, anthocyanins, and triterpenoids, have been demonstrated to inhibit COX-2 activity and prostanoid signaling [59,60,61,62]. Iridoids such as cornuside and morroniside inhibit prostaglandin synthesis in LPS-stimulated macrophages [59,63], while loganin downregulates NF-κB activation [64]. Consistent with interference with NF-κB signaling, iridoids downregulate proinflammatory cytokine expression in human skin fibroblasts and keratinocytes [2]. Anthocyanins and anthocyanidins, abundant in dark-fruit cultivars of European cornel such as ‘Uholok’ [16,61], also suppress COX-2 activity, though less potently than synthetic NSAIDs [65]. Notably, anthocyanidins lacking sugar moieties exhibit stronger antiproliferative effects than anthocyanins [66]. However, COX-2 modulation by anthocyanin-enriched extracts may vary over time, as shown in chokeberry-treated HT-29 cells where initial inhibition was followed by compensatory COX-2 upregulation [67], which was also reflected herein in the expression pattern of *mPGES1*.

Despite these observations, a systematic evaluation of cornel extract effects across multiple prostanoids in melanoma cell lines has been lacking. Our study demonstrates that cornel extracts modulate temporal prostanoid dynamics in a cell-type–specific and dose-dependent manner. In A375, low extract concentrations permitted continued temporal accumulation, particularly that of PGE_2_ and PGF_2α_, whereas higher concentrations flattened or reversed slopes. This pattern was most pronounced for Japanese cornel extract, rich in the iridoid glycosides morroniside, loganin, and sweroside. In contrast, extracts from European cornel cultivars (‘Uholok’ and ‘Yantarnyi’)—enriched in loganic acid and devoid of morroniside—exerted milder inhibitory effects, suggesting structural specificity among iridoids for regulation of proteins associated with a turnover of prostanoids. Inhibition of signaling pathways upstream of COX-2, such as NF-κB, ERK, and p38 MAPK, is a well-known mechanism of cornuside and loganin action [39,64]. Given the gene expression data presented in Section 2.3 and Section 2.4, the dose-dependent suppression of inducible prostanoids in A375 is unlikely to be mediated solely by downregulation of *PTGS2* transcripts, underscoring dissociation between transcriptional responses and short-term metabolic flux. In MeWo, temporal changes were smaller and only became appreciable at higher extract doses, reflecting the limited susceptibility of constitutive prostanoid synthesis to natural inhibitors. PGE_2_ and PGF_2α_ slopes, for example, remained near zero at low doses but became modestly negative at higher concentrations, indicating that only sustained or high-dose inhibition yields measurable suppression. The ‘Uholok’ cultivar, however, displayed unique behavior due to its anthocyanin content, which is absent in ‘Yantarnyi’ and Japanese cornel. Anthocyanins are potent antioxidants and modulators of inflammatory signaling [65]. They inhibit cancer cell proliferation, migration, and invasion by inducing apoptosis, arresting the cell cycle, and modulating pathways such as PI3K/Akt, MAPK, NF-κB, and apoptotic cascades. They downregulate oncogenes such as MYC while enhancing tumor suppressors such as TP53. Their representative, Cyanidin-3-O-glucoside, also suppresses inflammation by reducing COX-2, TNF-α, and IL-6, and induces cell cycle arrest and mitochondrial dysfunction in various cancers [68]. The stronger decline in PGF_2α_ observed in ‘Uholok’-treated A375 cells suggests that anthocyanins may synergize with iridoids to enhance suppression of COX-2–mediated pathways. By scavenging reactive oxygen species that serve as secondary activators of NF-κB and *PTGS2* transcription, anthocyanins could amplify the inhibitory potential of cornel phytochemicals. Taken together, the combined analysis of discrete concentration measurements (Section 2.1), continuous-time dynamic modeling (Section 2.2), and baseline gene expression profiles (Section 2.3) indicates that *Cornus* extracts primarily reshape prostanoid kinetics rather than simply reduce metabolite abundance at fixed time points.

Among prostanoids, PGE_2_ is the most extensively studied mediator of skin carcinogenesis, contributing to melanoma initiation, progression, angiogenesis, immune evasion, and metastasis [35,42,69,70]. Among others, PGE_2_ induces expression of indoleamine 2,3-dioxygenase (IDO)–1, a checkpoint molecule [71]. COX-2 inhibition has been found effective in reducing its mRNA and protein levels (reviewed in [21]). Overexpression of COX-2 and microsomal PGE_2_-specific synthase occurs in both melanoma [38] and supportive stromal cells within the tumor microenvironment [42,72]. It is accompanied by suppression of PGE_2_-degrading enzyme—15-prostaglandin dehydrogenase (reviewed in [35]). The inhibitory effect of cornel phytochemicals on COX-2 has been studied in the PGE_2_ context, while the potential effect on other COX-synthesized eicosanoids is unknown. PGF_2α_ is secreted by melanoma cells in abundance [26,73] and its expression is higher in melanomas than normal surrounding skin [74], but its role remains obscure. In normal melanocytes, PGF_2α_ facilitates formation of dendrites and pigmentation [73]. PGF_2α_ signals via FP receptors, present in normal melanocytes but detected in melanomas—primary or metastatic—neither at the mRNA nor protein level, implying a paracrine mode of action for PGF_2α_ [75]. In other cancers, PGF_2α_ activates Ras/ERK1/2 signaling and, like PGE_2_, is linked with growth promotion by stimulating cancer cell migration and neoangiogenesis (reviewed in [19]).

In contrast to PGE_2_ and PGF_2α_, PGD_2_ levels showed no dependence on extract concentration, implying that *Cornus* treatments did not directly affect its biosynthesis. Since PGD_2_ is often produced by hematopoietic or fibroblast-like cells and may exert anti-proliferative or pro-differentiation effects in the skin [21], the stability of its levels suggests selective targeting of tumor-promoting rather than homeostatic prostaglandins. However, downstream 15-deoxy-Δ^12^,^14^-PGJ_2_—a cyclopentenone derivative of PGD_2_ with PPARγ-activating and anti-inflammatory properties—responded to extract concentration, especially in A375 cells. Here, increasing doses of Japanese cornel extract led to a progressive flattening and eventual decline in 15-deoxy-Δ^12^,^14^-PGJ_2_ levels. Although this prostaglandin can suppress tumor growth, its accumulation is also a marker of oxidative stress [42]. Thus, moderate reduction may reflect restoration of redox balance rather than loss of anti-tumor signaling, although this interpretation remains speculative in the absence of direct redox measurements in this study. In MeWo cells, changes were less pronounced, consistent with their stable metabolic baseline. Again, variety-specific differences were evident: Japanese cornel exerted the strongest inhibitory influence, whereas the anthocyanin-rich ‘Uholok’ cultivar showed partial attenuation of this effect. This could suggest that anthocyanins, through antioxidant mechanisms, may stabilize PGD_2_ metabolism and reduce conversion to reactive downstream products like 15-deoxy-∆^12^,^14^-PGJ_2_. Such interplay between iridoid- and flavonoid-mediated regulation underscores the polypharmacological nature of *Cornus* extracts.

The temporal increase in 13,14-dihydro-PGE_1_ observed in untreated A375 cells was effectively neutralized by *Cornus* extracts. PGE_1_ exerts anti-inflammatory and vascular-protective effects by, respectively, downregulating IL-6 secretion [76] and reducing adhesion between endothelial cells and monocytes and downregulating adhesion molecules (VCAM-1, ICAM-1, and E-selectin) in endothelial cells. Mechanistically, PGE_1_ inhibits NF-κB activation and decreases reactive oxygen species production, thereby suppressing vascular inflammation [77]. However, it can also indicate ongoing prostaglandin turnover in tumor cells. The suppression of its rise by *Cornus* treatment suggests decreased upstream COX activity. As with other prostaglandins, the Japanese cornel extract produced the most consistent inhibition, whereas European cultivars had weaker effects, reinforcing the importance of morroniside, loganin, and sweroside as key active constituents. In MeWo cells, no meaningful temporal changes were detected, implying that metastatic cells’ prostanoid pools are less responsive to modulation of de novo synthesis, possibly due to post-translational regulation of COX enzymes or altered substrate flux.

For 6-keto-PGF_1α_, a stable metabolite of PGI_2_, and for LTB_4_, temporal trends were largely unaffected by extract concentration. PGI_2_ has an anti-inflammatory role in allergic reactions and acts by downregulating secretion of chemokines from circulating monocytes and airway epithelia [78]. Its activities are known to counterbalance these of thromboxane’s. PGI_2_ is implicated in regulating proliferation of immune cells, but the exact mechanisms are not known [79]. In cancer, the PGIS/PGI_2_ signaling pathway generally exhibits tumor-suppressive properties, with reduced PGIS expression correlating with poorer prognosis and increased tumor progression in several malignancies. Experimental evidence suggests that PGI_2_ and its analogs may inhibit tumor growth and metastasis through anti-inflammatory, antimitogenic, and PPAR-mediated mechanisms, highlighting their potential as therapeutic or chemopreventive agents [19]. Regarding PGI2’s role in melanoma, its presence has been shown to protect cancer cells from radiation stress [80]. The LTB_4_/BLT_1_ signaling regulates inflammation and immune cell recruitment in cancer, with effects varying by context. While it can enhance anti-tumor CD8^+^ T-cell infiltration and immune surveillance, activity of LTB_4_/BLT_1_ may also promote tumor progression through recruitment of immunosuppressive myeloid cells and neutrophils. In melanoma, LTB_4_-driven inflammation and BLT_1_-mediated leukocyte infiltration have been linked to increased metastasis and a tumor-promoting microenvironment, suggesting that blocking the LTB_4_–BLT_1_ axis could help limit melanoma progression [81]. The relative stability of PGI_2_ and LTB_4_ temporal trends suggests that *Cornus* extracts preferentially target COX-2–derived prostaglandins rather than broader eicosanoid metabolism. Given that PGI_2_ and LTB_4_ originate from distinct enzymatic branches (COX-1 and 5-LOX, respectively), their insensitivity is consistent with preferential modulation of COX-2–derived prostanoids. Minor variations observed between cornel varieties—particularly a weak modulation by the ‘Uholok’ cultivar—may reflect secondary antioxidant effects rather than direct enzymatic inhibition.

TXB_2_, a stable metabolite of thromboxane A_2_, displayed pronounced dose-dependent suppression in both melanoma lines, especially under treatment with Japanese cornel extract. Thromboxanes promote angiogenesis and metastasis [35], making their reduction an indicator of anti-metastatic potential. Elevated TXB_2_ and overexpression of its synthase and receptors are associated with multiple cancers, promoting proliferation, migration, angiogenesis, and metastasis. TXA_2_ signaling activates oncogenic pathways including PI3K/AKT, ERK, NF-κB, and Rho/ROCK, enhancing tumor cell survival and motility. Clinically, high TXB_2_ serves as a biomarker for poor prognosis, while TBXA_2_R blockade shows potential to inhibit tumor growth and metastasis [19]. In A375 cells, low extract concentrations initially allowed temporal increases, which were progressively flattened and reversed in the modeled temporal slopes at higher doses. MeWo cells showed a similar trend but with consistently lower baseline dynamics, again reflecting constitutive prostanoid homeostasis in metastatic cells. The attenuated dose response of the European cultivars, particularly ‘Yantarnyi’, implies that anthocyanins in ‘Uholok’ may contribute to thromboxane suppression through indirect oxidative mechanisms, while iridoids alone have a milder effect. Overall, the results point to significant inhibition of pro-angiogenic prostanoids by *Cornus* constituents.

### Study Limitations

The results obtained are promising, but it is important to mention the limitations of our study resulting from its pilot nature. With regard to the anticancer potential of cornel extracts, our preliminary results need to be supplemented with studies on their selectivity. It is also necessary to conduct detailed studies on their mechanism of action, as well as to isolate the components of the extract that exhibit activity modulating the eicosanoid synthesis pathway. In the absence of such information, the take-home message from this study is exploratory and hypothesis-generating rather than deterministic.

Moreover, there are limitations stemming from the used analytical framework. Time was sampled at three discrete points (24, 48, 72 h) for MS-associated measurements, which limits precise reconstruction of fine-grained temporal trajectories. Therefore, dynamic models estimate smoothed trends rather than instantaneous kinetic rates. Temporal effects were inferred using hierarchical statistical models rather than directly measured kinetic rates. While this allows integration of time-dependent trends and dispersion, it precludes interpretation in terms of absolute fluxes or per-unit-time biological rates. Notably, absolute metabolite concentrations at fixed time points do not fully capture the underlying temporal dynamics of prostanoids’ turnover. Differences between snapshot measurements (Section 2.1, Section 2.3 and Section 2.4) and modeled temporal effects are expected and reflect complementary, not conflicting, aspects of system behavior. Particularly, gene expression analyses were restricted to selected enzymes of prostanoids’ metabolism and provide information on transcriptional potential rather than enzymatic activity of post-translational regulation.

Above all, the experiments were conducted in melanoma cell lines under controlled in vitro conditions. Naturally, the insights from this study may not fully reflect the complexity of tumor micro-environments or in vivo regulation of prostanoids.

## 4. Materials and Methods

### 4.1. Cornel Plants and Preparation of Fruit Extracts

**Figure 9:**
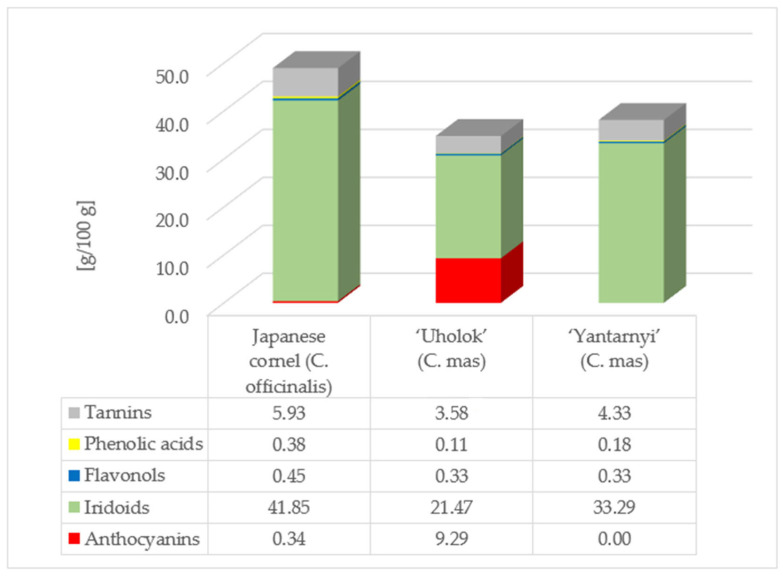
Contents (g/100 g dw) of iridoids and polyphenols in fruit extracts from Japanese (*C. officinalis*) and European (*C. mas*: ‘Uholok’ and ‘Yantarnyi’ cultivars) cornels. Dw, dry weight.

Arboretum and Institute of Physiography in Bolestraszyce, Poland provided plant material, authenticated by Prof. Narcyz Piórecki. This included ‘Yantarnyi’ and ‘Uholok’ cultivars of European cornel (*Cornus mas*) and Japanese cornel (*Cornus officinalis* Sieb. et Zucc.). The adequate voucher specimens (‘Yantarnyi’—BDPA 14131; ‘Uholok’—BDPA 14139; *Cornus officinalis* —BDPA 14020) have been deposited at the Herbarium of Arboretum in Bolestraszyce, Poland. Cornel fruits, fully ripe, were frozen at −20 °C immediately after harvesting and stored until further preparations. Cornel juice was obtained from defrosted fruits using a previously described method [58] and purified using chromatography on an Amberlite XAD-16 resin column (Rohm and Haas, Chauny Cedex, France). Column washing with distilled water was applied to remove organic impurities, while 80% ethanol (POCh, Gliwice, Poland) was used as an eluent. Obtained cornel extracts were subsequently concentrated at 40 °C under vacuum. Solvent was removed using a Rotavapor (Unipan, Warszawa, Poland) and the extracts were then freeze-dried (Alpha 1–4 LSC, Christ, Osterode am Harz, Germany). Composition and concentrations of chemicals in the obtained extracts were characterized using LC-MS and HPLC. The content of the studied extracts as well as qualitative and quantitative characterization of their main anthocyanins and iridoids is provided in Figure 9 and Table 9, respectively.

### 4.2. Cell Culture

Human melanoma cell lines—MeWo (ATCC^®^ HTB-65™) and A375 (ATCC^®^ CRL-1619™) were purchased from American Type Culture Collection (ATCC; Manassas, VA, USA). MeWo cells were cultured in Minimum Essential Medium (MEM) without phenol red (Gibco, Thermo Fisher Scientific, Waltham, MA, USA) supplemented with 2 mM GlutaMAX™ (Gibco), 1 mM sodium pyruvate solution (Sigma-Aldrich, Saint Louis, MO, USA), and MEM Non-Essential Amino Acid Solution (Sigma-Aldrich). A375 cells were cultured in Dulbecco’s Modified Eagle Medium (DMEM) without phenol red (Gibco). Cells were grown in culture flasks (T-75, Falcon^®^, Corning Life Sciences, Tewksbury, MA, USA) in the indicated culture media supplemented with 10% heat-inactivated fetal bovine serum (FBS) from Gibco and 1% stabilized antibiotic and antimycotic solution containing 10,000 units of penicillin/mL, 10 mg/mL of streptomycin, and 25 µg/mL of amphotericin B (Sigma-Aldrich). Medium was renewed every 2–3 days. Cells were cultured under standard conditions, that is, at 37 °C in humidified air containing 5% CO_2_ in a CELCULTURE^®^ CCL-170B-8 incubator (Esco Micro Pte Ltd., Singapore). For experiments or subculturing, cells were harvested with TrypLE™ Express (Gibco), stained with 0.4% trypan blue solution, and counted using a Burker chamber.

For metabolomic analysis, cells were seeded at 3 × 10^5^ cells/well in a 6-well plate. After seeding, cells were maintained for 24 h in a CO_2_ incubator for cell attachment and homeostasis. Next, the culture medium was withdrawn from the wells and replaced with 1.5 mL of its fresh portion with the addition of indicated concentrations of fruit extracts from Japanese cornel (*Cornus officinalis* Sieb. & Zucc.) and from ‘Uholok’ and ‘Yantarnyi’ cultivars of European cornel (*Cornus mas*). Stock aqueous extract solutions (10 mg/mL) were used to prepare exposure concentrations of 100, 250, and 500 µg/mL for both cell lines and additionally 750 µg/mL for MeWo cells. This experiment was performed in three biological replicates, utilizing cells from different cell passages. Cells treated with culture medium without the extract addition were used as the respective controls. Extract concentrations were selected based on previous findings [58] and results of the cell viability assay using sulforhodamine B (SRB) presented in Supplementary Materials Figures S1–S6. 

Following media removal after 24, 48, and 72 h, cells were washed twice with PBS and subjected to extraction with 70% (*v*/*v*) cold methanol in water. Methanol extracts were then collected and stored at −80 °C until LC-MS/MS analysis. Prior to measurements, samples were dried using a centrifugal vacuum concentrator (Hetovac from Heto Lab Equipment, Roskilde, Denmark) at 50 °C and then dissolved in water to allow for protein concentration determination using the Bradford method with Pierce™ Bradford Protein Assay Kit (ThermoScientific, Waltham, MA, USA) against a standard curve based on bovine serum albumin.

For the purpose of the gene expression study, cells were prepared, treated, and cultured as described above, except for they were lysed with 1 mL of TRIzol reagent (Invitrogen, Thermo Fisher Scientific, Waltham, MA, USA) and stored at −80 °C until RNA extraction and RT-qPCR analysis.

### 4.3. Quantifying the Concentration of Prostanoids and LTB_4_ with LC-MS (Targeted Metabolomic Analysis)

Standards of Thromboxane B_2_, Leukotriene B_4_, Prostaglandin D_2_, Prostaglandin E_2_, 6-keto Prostaglandin F_1α_, Prostaglandin F_2α_, 15-deoxy-Δ^12^,^14^-Prostaglandin J_2_, 13,14-dihydro Prostaglandin E_1_, and their isotope-labeled standards were procured from Cayman Chemical Company (Ann Arbor, MI, USA). Toluene, methyl tert-butyl ether (MTBE), and formic acid (FA) were acquired from Merck Millipore (Warsaw, Poland). Methanol, acetonitrile (ACN), ethyl acetate, water, and formic acid (FA) were acquired from Witko (Warsaw, Poland).

Compounds were separated using a triple quadrupole mass spectrometer (Xevo Absolute from Waters Corp., Milford, MA, USA) and a previously described method [82]. Briefly, cell lysates were obtained by washing the cells twice with PBS and then adding ice-cold 70% methanol. A 1 mL aliquot of the obtained supernatant was evaporated to dryness and re-dissolved in 25 µL of internal standards in 20% ACN in water and transferred before analysis into autosampler glass vials.

Chromatographic separation of metabolites was conducted on a BEH Shield C18 column (100 mm × 2.1 mm i.d., 1.7 µm; Waters). Data acquisition for all compounds was carried out on MassLynx Software 10.50 (Waters) in multiple reaction monitoring mode (MRM).

### 4.4. RNA Extraction and RT-qPCR Analysis

#### 4.4.1. RNA Extraction and Purification

An aliquot of 100 μL of β-mercaptoethanol (Sigma-Aldrich, St. Luis, MO, USA) was added to cell lysates, and phenol–chloroform RNA extraction was performed and supplemented with the spin column method purification using a PureLink™ RNA Mini Kit (Invitrogen). Genomic DNA contamination was avoided by on-column digestion with DNAse (PureLink™ DNase Set, Invitrogen). Quantity, quality, and integrity assessments of RNA isolates were conducted using a NanoDrop 2000 spectrophotometer (Thermo-Fisher Scientific) and a LabChip microfluidic technology on the Experion platform with Experion RNA StdSens analysis kits (BioRad, Hercules, CA, USA).

#### 4.4.2. cDNA Synthesis

cDNAs were synthesized from 1 μg of RNA using an iScript™ cDNA Synthesis Kit (BioRad) and a C1000 thermocycler (BioRad) following the manufacturer’s instructions.

#### 4.4.3. qPCR Analysis

Following five-fold dilution, the obtained cDNAs served as templates (1:10, *v*/*v*) in qPCR conducted using 2×SsoFast EvaGreen^®^ Supermix (BioRad) and a CFX96 Real-Time PCR system (BioRad) as per the manufacturer’s instructions. Reaction conditions were set as follows: polymerase activation—30 s at 95 °C, 40 cycles comprising denaturation—5 s at 95 °C and annealing and extension—5 s at 61 °C. Reaction products were analyzed to confirm their specificity by melting curve analysis (60–95 °C, read every 0.5 °C) and agarose (SeaKem LE agarose)/SYBR Green electrophoresis (Lonza, Basel, Switzerland). Intron-spanning primers (Table 10) were generated by Genomed (Warsaw, Poland) based on sequences proposed by OriGene Technologies, Inc. (Rockville, MD, USA; www.origene.com, assessed on 17 October 2024).

The Cq values of technical replicates were averaged, and geometric means for all samples for each gene of interest were calculated. The Cqs of the individual samples were subsequently subtracted from the geometric means, and the obtained ΔCq values were linearized by 2^ΔCq^ conversion, followed by normalization to *GAPDH* serving as a reference gene. The values calculated are referred to as a “normalized relative quantity” (NRQ) [83] and subjected to statistical analysis.

### 4.5. Cell Viability Determination

Cell viability was determined using the modified sulforhodamine B (SRB) assay as previously described [58]. For the purpose of present study, cells (A375 and MeWo) were treated with cornel extracts (*C. officinalis*, *C. mas* ‘Uholok’, and *C. mas* ‘Yantarnyi’) in concentrations of 0 (controls; untreated cells used as reference), 2.5, 10, 25, 50, 100, 200, 300, 500 and 750 μg/mL for 6, 12, 24, 48, and 72 h. Each measurement was conducted in five technical replicates, which were averaged prior to analysis. Each experiment was conducted three times. Results of these biological replicates were subjected to statistical analysis as described in Section 4.6.8.

### 4.6. Data Analysis

To dissect the temporal evolution of prostanoids and LTB_4_ production in melanoma cells under experimental modulation, we modeled each of the eight measured metabolites independently. Recognizing that omics-derived concentration data often deviate substantially from classical assumptions of normality—displaying heteroskedasticity, heavy tails, and skewness—we employed a distributionally flexible approach using Generalized Additive Models for Location, Scale, and Shape (GAMLSS) [84]. Each metabolite was modeled assuming a Generalized Gamma distribution (μ, σ, ν), which estimates not only the central location (*mu*) but also dispersion (*sigma*) and skewness (*nu*) parameters. This allowed the models to adapt to the empirical complexity of the data, while offering interpretability for each statistical dimension. The entire analysis was done in R 4.5.0.

#### 4.6.1. Model Architecture

The location parameter (mu) was modeled using an initially comprehensive formula designed to capture both main effects and biologically plausible interactions:

mu = timepoint_scaled + celltype + cornustype + cornusconc_scaled + timepoint_scaled:cornustype + timepoint_scaled:celltype + timepoint_scaled:cornusconc_scaled + timepoint_scaled:celltype:cornusconc_scaled + timepoint_scaled:celltype:cornustype + timepoint_scaled:cornustype:cornusconc_scaled + replicationmu = timepoint_scaled + celltype + cornustype + cornusconc_scaled + timepoint_scaled:cornustype + timepoint_scaled:celltype + timepoint_scaled:cornusconc_scaled + timepoint_scaled:celltype:cornusconc_scaled + timepoint_scaled:celltype:cornustype + timepoint_scaled:cornustype:cornusconc_scaled + replication

Here, timepoint_scaled represents the scaled time variable indicating progression of cell culture; celltype denotes melanoma cell line identity (A375 or MeWo); cornustype refers to the cornel variety applied (European cornel ‘Uholok’ (black) and ‘Yantarnyi’ (yellow) or Japanese cornel); and cornusconc_scaled captures the scaled dose of cornel extract. The variable replication controls for batch-specific variation. Although zero values were present in the dataset, variables were standardized (using mean-centering and dividing by the standard deviation) instead of setting 0 as the reference. This approach reduces standard error inflation in interaction terms and improves numerical stability, especially with asymmetrically distributed continuous variables.

Model specification for the mu component was iteratively refined through backward elimination of non-significant high-order interactions to improve parsimony, stabilize estimation, and enhance biological interpretability. The final models retained only those terms contributing meaningfully to the explanation of variance in prostaglandin dynamics. For the sigma and nu parameters, only significant main effects were retained, as their primary function was to correct for non-constant variance and asymmetry rather than to serve as primary variables of inference.

#### 4.6.2. Fixed vs. Random Effects: Treatment of Replication

Although replication could theoretically be modeled as a random effect to account for repeated measures or a nested experimental design, this approach was not feasible given the small number of replication levels (k = 3). In mixed models, reliable estimation of random effects variance components requires a sufficiently large number of clusters or grouping levels (typically ≥5–6). With only three replicates, variance estimates would be unstable and prone to overfitting or convergence failure. Therefore, replication was instead modeled as a fixed effect, allowing the analysis to control for potential batch-to-batch variation without introducing unreliable random structure.

This choice is methodologically sound and commonly recommended in small-sample scenarios, as fixed effects provide a conservative yet stable adjustment for systematic inter-replicate differences without requiring the estimation of random-effect variance from insufficient data.

#### 4.6.3. Handling Zero-Inflated Outcomes

In the case of PGD_2_, the concentration values included a substantial proportion of zeros. To account for zero inflation, we modeled this metabolite using a zero-inflated Gamma distribution via the glmmTMB framework. While this model does not allow joint estimation of dispersion or skewness (a limitation), it offers the necessary flexibility to handle structural zeros—something not supported by GAMLSS. The use of ziGamma was thus a pragmatic compromise, trading shape modeling for the accurate handling of non-detects.

#### 4.6.4. Model Diagnostics

Diagnostic evaluation of the GAMLSS models was performed via residual and quantile plots (plot() function in R) to assess goodness of fit and the adequacy of the assumed distribution. For the glmmTMB model, residual diagnostics were conducted using DHARMa, a contemporary [85] simulation-based approach designed to detect misspecification in mixed and zero-inflated models, including deviations from distributional assumptions, overdispersion, and zero-inflation misspecification.

#### 4.6.5. Estimation Focus: Simple Effects of Time

Although interaction terms were incorporated into the initial models to capture complex dependencies, the final inference was centered on estimating the simple effect of time (timepoint_scaled) under specific experimental conditions. This was done to disentangle whether a statistically significant interaction indeed reflected a meaningful directional trend in prostaglandin levels over time. Such simple effects provide biologically actionable insight into whether a given treatment or cell context accelerates, suppresses, or alters the temporal dynamics of prostaglandin production.

#### 4.6.6. Rationale for Methodological Strategy and Translational Relevance

This modeling approach was deliberately chosen to bridge statistical rigor with biological realism, offering a flexible yet interpretable framework to understand how melanoma cells respond dynamically to experimental perturbations. While classical factorial analyses (e.g., ANOVA-like contrasts) emphasize static comparisons across treatment groups, they lack the resolution to capture how a biological system evolves over time under compound stimuli. In contrast, time-aware modeling reflects the fact that tumor biology is dynamic, not dichotomous. The tumor microenvironment, cell-treatment interactions, and mediator synthesis (such as prostaglandins) all exhibit temporal dependencies that cannot be meaningfully addressed by endpoint-only analyses.

From a translational perspective, modeling prostaglandin kinetics over time rather than comparing fixed treatment conditions more closely mimics the progression of tumor growth and inflammation in vivo. In clinical settings, the timing and duration of biochemical responses are critical determinants of therapeutic success. By capturing not only whether but when prostaglandin levels shift in response to treatment, this approach provides mechanistic insights with potential relevance for scheduling, dosing, and therapeutic window optimization in anti-inflammatory or anti-tumor interventions.

Moreover, the use of distributionally flexible models like GAMLSS acknowledges and embraces the heterogeneity and asymmetry inherent to biological data, especially in cancer systems, where metabolic flux is not uniformly regulated. It supports a move away from oversimplified statistical assumptions toward approaches better aligned with the complex and nonlinear nature of tumor biology.

In sum, this strategy does not merely quantify treatment effects—it reconstructs time-resolved biological trajectories in a statistically robust manner, offering a blueprint for future metabolomic studies aimed at mechanistic discovery and clinical translation.

#### 4.6.7. Statistical Analysis of Baseline Metabolite Concentrations and Gene Expression

Normality of distribution was tested using the Kolmogorov–Smirnov test and homogeneity of variances using the Levene test. Data were analyzed using the Kruskal–Wallis *H* test with Conover post hoc test and with Jonckheere-Terpstra trend test in the case of multigroup comparisons and with *t*-test for independent samples with Welch correction for two-group comparisons. These analyses were conducted using MedCalc^®^ Statistical Software version 23.3.7 (MedCalc Software Ltd., Ostend, Belgium; https://www.medcalc.org; 2025) licensed to Prof. Malgorzata Krzystek-Korpacka. Statistical significance was set at probability (*p*) < 0.05, and all calculated *p*-values were two-tailed.

#### 4.6.8. Statistical Analysis of Cell Viability Results

Normality of distribution was tested using the Kolmogorov–Smirnov test and homogeneity of variances using the Levene test. Data were analyzed using the Kruskal–Wallis *H* test with the Conover *post hoc* test and with the Jonckheere-Terpstra trend test in the case of time-effect evaluation and with the Mann–Whitney U test for cell type-effect evaluation. These analyses were conducted using MedCalc^®^ Statistical Software version 23.6.2 (MedCalc Software Ltd, Ostend, Belgium; https://www.medcalc.org; 2026) licensed to Prof. Malgorzata Krzystek-Korpacka. Statistical significance was set at probability (*p*) < 0.05, and all calculated *p*-values were two-tailed.

## 5. Conclusions

In summary, *Cornus* extracts modulate melanoma prostanoid metabolism in a cell line–, dose–, and variety–specific manner. The Japanese cornel, enriched in morroniside, loganin, and sweroside, exerts strong inhibition of pro-tumorigenic prostaglandins PGE_2_, PGF_2α_, and TXB_2_, in a manner consistent with COX-2-targeting mechanisms described for its iridoids. European cornel cultivars, particularly the anthocyanin-rich ‘Uholok’, display milder but still consistent effects, reflecting combined antioxidant and anti-inflammatory actions. These differences underline the importance of phytochemical composition in determining biological efficacy. The selective targeting of COX-2–linked prostanoid pathways, coupled with the low toxicity expected of natural metabolites, highlights *Cornus* as a relevant source of bioactive compounds for modulating prostanoid-driven inflammatory signaling in melanoma. Further mechanistic and translational studies are warranted to validate their clinical potential and explore synergistic use with existing therapies.

## Figures and Tables

**Table 1:** Estimates of time-dependent effects for PGE_2_.

Effect	Interpretation	*β_i_*	*β_i_* SE	*t*	*p*
time	A. Change in metabolite conc. over time in A375 cells exposed to Japanese cornel	1.209	0.184	6.581	<0.001
time × varietyU	B. Change in A. upon exposure to ‘Uholok’	−0.537	0.258	−2.084	0.038
time × varietyY	C. Change in A. upon exposure to ‘Yantarnyi’	−0.743	0.265	−2.805	0.005
time × cellsM	D. Change in A. between MeWo and A375 cells	−1.356	0.200	−6.773	<0.001
time × extract	E. Change in A. with an increase in extract conc.	−0.300	0.062	−4.845	<0.001
time × cellsM × varietyU	Change in D. between exposure to ‘Uholok’ and Japanese cornel …or… Change in B. between MeWo and A375 cells	0.659	0.293	2.250	0.025
time × cellsM × varietyY	Change in D. between exposure to ‘Yantarnyi’ and Japanese cornel …or… Change in C. between MeWo and A375 cells	0.883	0.293	3.014	0.003

The effects describing temporal metabolite dynamics were estimated using generalized additive models for location, scale, and shape (GAMLSS). The parameter *βᵢ* represents the estimated effect of the predictor on the temporal dynamics of metabolite concentration, with associated standard error (SE), *t*-statistic, and *p*-value. All effects are reported on the link scale (log). Effects are considered significant if *p* < 0.05. Conc., concentration; time, timepoint_scaled; cellsM, celltypeMeWo; extract, extract concentration (cornusconc_scaled); varietyY, cornustypeYellow_fruit (‘Yantarnyi’); varietyU, cornustypeBlack_fruit (‘Uholok’).

**Table 2:** Estimates of time-dependent effects for PGF_2α_.

Effect	Interpretation	*β_i_*	*β_i_* SE	*t*	*p*
time	A. Change in metabolite conc. over time in A375 cells exposed to Japanese cornel	0.713	0.146	4.869	<0.001
time × varietyU	B. Change in A. upon exposure to ‘Uholok’	−0.542	0.215	−2.519	0.012
time × varietyY	C. Change in A. upon exposure to ‘Yantarnyi’	−0.469	0.198	−2.371	0.019
time × cellsM	D. Change in A. between MeWo and A375 cells	−0.849	0.160	−5.318	<0.001
time × extract	E. Change in A. with an increase in extract conc.	−0.527	0.117	−4.503	<0.001
time × cellsM × extract	Change in D. with an increase in extract conc. …or… Change in E. between MeWo and A375 cells	0.412	0.128	3.219	0.001
time × cellsM × varietyU	Change in D. between exposure to ‘Uholok’ and Japanese cornel …or… Change in B. between MeWo and A375 cells	0.481	0.228	2.110	0.036
time × cellsM × varietyY	Change in D. between exposure to ‘Yantarnyi’ and Japanese cornel …or… Change in C. between MeWo and A375 cells	0.491	0.213	2.300	0.022

The effects describing temporal metabolite dynamics were estimated using generalized additive models for location, scale, and shape (GAMLSS). The parameter *βᵢ* represents the estimated effect of the predictor on the temporal dynamics of metabolite concentration, with associated standard error (SE), *t*-statistic, and *p*-value. All effects are reported on the link scale (log). Effects are considered significant if *p* < 0.05. Conc., concentration; time, timepoint_scaled; cellsM, celltypeMeWo; extract, extract concentration (cornusconc_scaled); varietyY, cornustypeYellow_fruit (‘Yantarnyi’); varietyU, cornustypeBlack_fruit (‘Uholok’).

**Table 3:** Estimates of time-dependent effects for PGD_2_.

Effect	Interpretation	*β_i_*	*β_i_* SE	*t*	*p*
time	A. Change in metabolite conc. over time in A375 cells exposed to Japanese cornel	0.793	0.154	5.140	<0.001
time × cellsM	B. Change in A. between MeWo and A375 cells	−1.395	0.207	−6.740	<0.001
time × varietyU	C. Change in A. upon exposure to ‘Uholok’	−0.563	0.241	−2.330	0.020
time × varietyY	D. Change in A. upon exposure to ‘Yantarnyi’	−0.698	0.241	−2.890	0.004
time × cellsM × varietyU	Change in B. between exposure to ‘Uholok’ and Japanese cornel …or… Change in C. between MeWo and A375 cells	0.787	0.307	2.560	0.010
time × cellsM × varietyY	Change in B. between exposure to ‘Yantarnyi’ and Japanese cornel …or… Change in D. between MeWo and A375 cells	0.870	0.308	2.820	0.005

The effects describing temporal metabolite dynamics were estimated using a zero-inflated Gamma distribution. The parameter *βᵢ* represents the estimated effect of the predictor on the temporal dynamics of metabolite concentration, with associated standard error (SE), *t*-statistic, and *p*-value. All effects are reported on the link scale (log). Effects are considered significant if *p* < 0.05. Conc., concentration; time, timepoint_scaled; cellsM, celltypeMeWo; extract, extract concentration (cornusconc_scaled); varietyY, cornustypeYellow_fruit (‘Yantarnyi’); varietyU, cornustypeBlack_fruit (‘Uholok’).

**Table 4:** Estimates of time-dependent effects for 15-deoxy-Δ^12^,^14^-PGJ_2_.

Effect	Interpretation	*β_i_*	*β_i_* SE	*t*	*p*
time	A. Change in metabolite conc. over time in A375 cells, exposed to Japanese cornel	0.226	0.119	1.900	0.059
time × varietyU	B. Change in A. upon exposure to ‘Uholok’	−0.177	0.100	−1.778	0.077
time × varietyY	C. Change in A. upon exposure to ‘Yantarnyi’	−0.187	0.106	−1.758	0.080
time × cellsM	D. Change in A. between MeWo and A375 cells	−0.144	0.083	−1.733	0.084
time × extract	E. Change in A. with an increase in extract conc.	−0.316	0.099	−3.191	0.002
time × cellsM × extract	Change in D. with an increase in extract conc. …or… Change in E. between MeWo and A375 cells	0.164	0.079	2.062	0.040
time × varietyU × extract	Change in B. with an increase in extract conc. …or… Change in E. between exposure to ‘Uholok’ and Japanese cornel	0.152	0.082	1.857	0.065
time × varietyY × extract	Change in C. with an increase in extract conc. …or… Change in E. between exposure to ‘Yantarnyi’ and Japanese cornel	0.185	0.085	2.167	0.031

The effects describing temporal metabolite dynamics were estimated using generalized additive models for location, scale, and shape (GAMLSS). The parameter *βᵢ* represents the estimated effect of the predictor on the temporal dynamics of metabolite concentration, with associated standard error (SE), *t*-statistic, and *p*-value. All effects are reported on the link scale (log). Effects are considered significant if *p* < 0.05. Conc., concentration; time, timepoint_scaled; cellsM, celltypeMeWo; extract, extract concentration (cornusconc_scaled); varietyY, cornustypeYellow_fruit (‘Yantarnyi’); varietyU, cornustypeBlack_fruit (‘Uholok’).

**Table 5:** Estimates of time-dependent effects for 13,14-dihydro-PGE_1_.

Effect	Interpretation	*β_i_*	*β_i_* SE	*t*	*p*
time	A. Change in metabolite conc. over time in A375 cells	−0.038	0.028	−1.339	0.182
time × cellsM	B. Change in A. between MeWo and A375 cells	0.038	0.031	1.213	0.226
time × extract	C. Change in A. with an increase in extract conc.	−0.124	0.035	−3.495	0.001
time × cellsM × extract	Change in B. with an increase in extract conc. …or… Change in C. between MeWo and A375 cells	0.119	0.037	3.223	0.001

The effects describing temporal metabolite dynamics were estimated using generalized additive models for location, scale, and shape (GAMLSS). The parameter *βᵢ* represents the estimated effect of the predictor on the temporal dynamics of metabolite concentration, with associated standard error (SE), *t*-statistics, and *p*-value. All effects are reported on the link scale (log), representing a fold-change. Effects are considered significant if *p* < 0.05. Conc., concentration; time, timepoint_scaled; cellsM, celltypeMeWo; extract, extract concentration/dose (cornusconc_scaled).

**Table 6:** Estimates of time-dependent effects for 6-keto-PGF_1α_.

Effect	Interpretation	*β_i_*	*β_i_* SE	*t*	*p*
time	A. Change in metabolite conc. over time in A375 cells exposed to Japanese cornel	0.452	0.098	4.607	<0.001
time × varietyU	Change in A. upon exposure to ‘Uholok’	−0.251	0.120	−2.093	0.037
time × varietyY	Change in A. upon exposure to ‘Yantarnyi’	0.009	0.115	0.079	0.937
time × cellsM	Change in A. between MeWo and A375 cells	−0.458	0.101	−4.517	<0.001

The effects describing temporal metabolite dynamics were estimated using generalized additive models for location, scale, and shape (GAMLSS). The parameter *βᵢ* represents the estimated effect of the predictor on the temporal dynamics of metabolite concentration, with associated standard error (SE), *t*-statistic, and *p*-value. All effects are reported on the link scale (log). Effects are considered significant if *p* < 0.05. Conc., concentration; time, timepoint_scaled; cellsM, celltypeMeWo; varietyY, cornustypeYellow_fruit (‘Yantarnyi’); varietyU, cornustypeBlack_fruit (‘Uholok’).

**Table 7:** Estimates of time-dependent effects for TXB_2_.

Effect	Interpretation	*β_i_*	*β_i_* SE	*t*	*p*
time	A. Change in metabolite conc. over time in A375 cells exposed to Japanese cornel	0.246	0.176	1.393	0.165
time × varietyU	B. Change in A. upon exposure to ‘Uholok’	0.405	0.217	1.867	0.063
time × varietyY	C. Change in A. upon exposure to ‘Yantarnyi’	0.552	0.351	1.570	0.118
time × cellsM	D. Change in A. between MeWo and A375 cells	−0.257	0.185	−1.387	0.167
time × extract	E. Change in A. with an increase in extract conc.	−0.280	0.058	−4.801	<0.001
time × cellsM × varietyU	Change in D. between exposure to ‘Uholok’ and Japanese cornel …or… Change in B. between MeWo and A375 cells	−0.445	0.231	−1.925	0.056
time × cellsM × varietyY	Change in D. between exposure to ‘Yantarnyi’ and Japanese cornel …or… Change in C. between MeWo and A375 cells	−0.552	0.354	−1.558	0.121
time × varietyU × extract	Change in B. with an increase in extract conc. …or… Change in E. between exposure to ‘Uholok’ and Japanese cornel	0.226	0.090	2.520	0.013
time × varietyY × extract	Change in C. with an increase in extract conc. …or… Change in E. between exposure to ‘Yantarnyi’ and Japanese cornel	0.162	0.086	1.878	0.062

The effects describing temporal metabolite dynamics were estimated using generalized additive models for location, scale, and shape (GAMLSS). The parameter *βᵢ* represents the estimated effect of the predictor on the temporal dynamics of metabolite concentration, with associated standard error (SE), *t*-statistic, and *p*-value. All effects are reported on the link scale (log). Effects are considered significant if *p* < 0.05. Conc., concentration; time, timepoint_scaled; cellsM, celltypeMeWo; extract, extract concentration (cornusconc_scaled); varietyY, cornustypeYellow_fruit (‘Yantarnyi’); varietyU, cornustypeBlack_fruit (‘Uholok’).

**Table 8:** Estimates of time-dependent effects for LTB_4_.

Effect	Interpretation	*β_i_*	*β_i_* SE	*t*	*p*
time	A. Change in metabolite conc. over time in A375 cells, exposed to Japanese cornel	−0.673	0.172	−3.907	<0.001
time × varietyU	B. Change in A. upon exposure to ‘Uholok’	0.835	0.179	4.658	<0.001
time × varietyY	C. Change in A. upon exposure to ‘Yantarnyi’	0.918	0.180	5.092	<0.001
time × cellsM	D. Change in A. between MeWo and A375 cells	0.774	0.173	4.464	<0.001
time × extract	E. Change in A. with an increase in extract conc.	−0.070	0.046	−1.524	0.129
time × cellsM × varietyU	Change in D. between exposure to ‘Uholok’ and Japanese cornel …or… Change in B. between MeWo and A375 cells	−1.022	0.186	−5.497	<0.001
time × cellsM × varietyY	Change in D. between exposure to ‘Yantarnyi ‘ and Japanese cornel …or… Change in C. between Mewo and A375 cells	−1.158	0.186	−6.213	<0.001
time × varietyU × extract	Change in B. with an increase in extract conc. …or… Change in E. between exposure to ‘Uholok’ and Japanese cornel	0.125	0.063	2.002	0.047
time × varietyY × extract	Change in C. with an increase in extract conc. …or… Change in E. between exposure to ‘Yantarnyi’ and Japanese cornel	0.063	0.061	1.031	0.304

The effects describing temporal metabolite dynamics were estimated using generalized additive models for location, scale, and shape (GAMLSS). The parameter *βᵢ* represents the estimated effect of the predictor on the temporal dynamics of metabolite concentration, with associated standard error (SE), *t*-statistic, and *p*-value. All effects are reported on the link scale (log). Effects are considered significant if *p* < 0.05. Conc., concentration; time, timepoint_scaled; cellsM, celltypeMeWo; extract, extract concentration (cornusconc_scaled); varietyY, cornustypeYellow_fruit (‘Yantarnyi’); varietyU, cornustypeBlack_fruit (‘Uholok’).

**Table 9:** Qualitative and quantitative characterization of main anthocyanins and iridoids in fruit extracts from Japanese (*C. officinalis*) and European (*C. mas*: ‘Uholok’ and ‘Yantarnyi’ cultivars) cornels.

Compound	Quality (*m*/*z*)		Quantity (g/100 g dw)		
	MS	MS/MS	Japanese Cornel (*C. officinalis*)	European Cornel (*C. mas*)	
				‘Uholok’	‘Yantarnyi’
Anthocyanins:	Positive Ion Mode				
Cyanidin 3-*O*-galactoside	449 [M + H]^+^	287	0.01	4.83	ni
Pelargonidin 3-*O*-galactoside	433 [M + H]^+^	271	0.10	2.12	ni
Iridoids:	Negative Ion Mode				
Loganic acid	375 [M − H]^−^	213	3.83	19.66	29.14
Morroniside	405 [M − H]^−^ 451 [M − H + HCOO]^−^	243	25.65	ni	ni
Loganin	389 [M − H]^−^ 435 [M − H + HCOO]^−^	227	9.33	tr	1.91

Dw, dry weight; *m*/*z*, mass-to-charge ratio; MS, mass spectrometry—a single mass analysis; MS/MS, mass analysis involving fragmentation for structural analysis—masses of parents and their fragments; ni: not identified; tr: trace.

**Table 10:** Primers used in the study.

Symbol	Encoded Protein	Primers’ Sequences		Size [bp]
		Forward	Reverse	
*GAPDH*	Glyceraldehyde-3-phosphate dehydrogenase	gtctcctctgacttcaacagcg	accaccctgttgctgtagccaa	131
*PTGS2*	Prostaglandin-endoperoxide synthase 2 (COX2)	cggtgaaactctggctagacag	gcaaaccgtagatgctcaggga	156
*mPGES1*	Microsomal prostaglandin E synthase	gaggatgccctgagacacgga	ccagaaaggagtagacgaagcc	136
*HPGD*	15-hydroxyprostaglandin dehydrogenase 1	tggaggtgaaggcggcatcatt	gagcgtgtgaatccaactatgcc	114

bp, base pairs.

## Data Availability

The datasets presented in this article are not readily available because the datasets are not publicly available, they are part of an ongoing institutional research project, and their dissemination is subject to the data management polices of the institution. Requests to access the datasets should be directed to from the corresponding author upon reasonable request and with the permission of the institution.

## Supplementary Materials

The following supporting information can be downloaded at: https://www.mdpi.com/article/10.3390/ijms27156982/s1.

## Abbreviations

The following abbreviations are used in this manuscript:

ACN	Acetonitrile
AKR1C3	Aldo-Keto Reductase Family 1 Member C3
AKT	Protein kinase B (PKB)
CI	Confidence interval
Conc.	Concentration
COX	Cyclooxygenase (protein)
DHARMa	Residual Diagnostics for Hierarchical (Multi-Level/Mixed) Regression Models
EP	Prostaglandin E_2_ Receptor
ERK	Extracellular signal-regulated kinase
FA	Formic Acid
GAMLSS	Generalized Additive Models for Location, Scale, and Shape
GAPDH	Glyceraldehyde-3-phosphate dehydrogenase
*HPGD*	15-hydroxyprostaglandin dehydrogenase 1
IQR	Interquartile range
LC-MS	Liquid Chromatography—Mass Spectrometry
LPS	Lipopolysaccharide
LT	Leukotriene
*mPGES1*	Microsomal Prostaglandin E Synthase
MTBE	Methyl tert-Butyl Ether
NF-κB	Nuclear Factor kappa-light-chain-enhancer of activated B cells
NRQ	Normalized relative quantity
NSAIDs	Nonsteroidal anti-inflammatory drugs
PD-1	Programmed death 1
PD-L1	Programmed death ligand 1
PG	Prostaglandin
PI3K	Phosphatidylinositol 3-kinase
*PTGS2*	Cyclooxygenase 2 (gene)
ROCK	Rho-associated protein kinase
SE	Standard error
TX	Thromboxane
