# Supplementary material for: Time-Resolved Metabolomics Reveals Distinct, Cell- and Variety-Dependent Profiles of Prostanoids in Melanoma Cells Exposed to Fruit Extracts from Cornus mas and C. officinalis: A Pilot Study
Source: Int J Mol Sci. 2026 Aug 3;27(15):6982. doi: 10.3390/ijms27156982 (PMC13467092; doi:10.3390/ijms27156982)
Supplement: Supplementary file 1 [file ijms-27-06982-s001.zip › Figure S1.pdf]

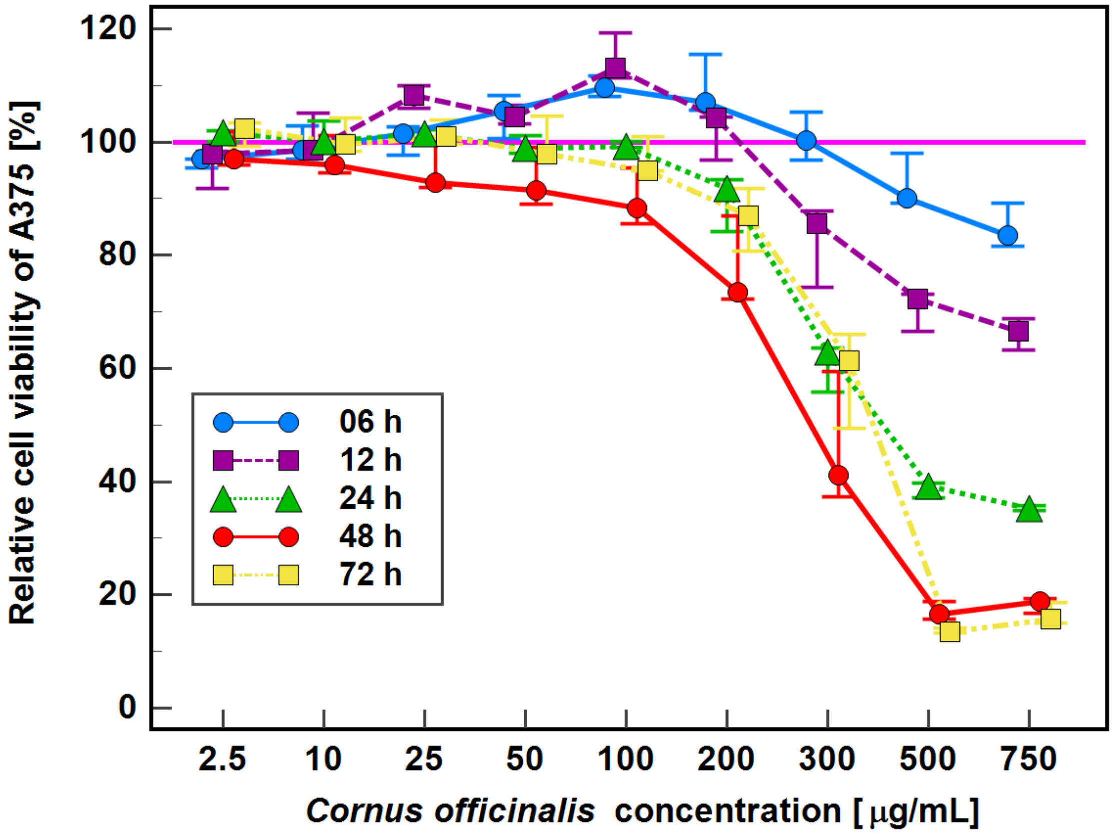

Figure S1. Viability of A375 cells treated for 6-72h with varying concentrations of *Cornus officinalis* concentrations (2.5-750 µg/mL) relative to untreated cells assessed using SRB assay and expressed in [%].

Data presented as medians (circles, squares, and triangles) with interquartile range (whiskers) and analyzed using Kruskal-Wallis H test with Conover post-hoc test and Jonckheere-Terpstra trend test. SRB assay methodology as described in [58].

Accompanying statistics:

Data: relative cell viability [%] for A375 cells at 6h
Factor: concentration of *Cornus officinalis* [µg/mL]

Data	survival%
Factor	conc.
Filter	time="06 h"

Sample size	27

Descriptive statistics

Factor	n	Minimum	25th percentile	Median	75th percentile	Maximum
2.5	3	94.8299	95.376	97.015	97.049	97.060
10	3	96.3804	96.921	98.542	102.948	104.416
25	3	96.3117	97.628	101.576	102.649	103.007
50	3	98.8868	100.550	105.538	108.314	109.239
100	3	107.5501	108.073	109.642	111.754	112.458
200	3	105.1810	105.658	107.090	115.509	118.316
300	3	95.7061	96.834	100.217	105.223	106.892
500	3	88.9224	89.219	90.110	97.991	100.619
750	3	80.8989	81.557	83.533	89.267	91.178

Kruskal-Wallis test

Test statistic	19.6614
Corrected for ties	19.6614
Degrees of Freedom (DF)	8
Significance level	P = 0.011696

Post-hoc analysis (Conover)

Factor	n	Average Rank	Different (P<0.05) from factor nr
(1) 2.5	3	9.00	(4)(5)(6)
(2) 10	3	13.00	(5)(6)(9)
(3) 25	3	13.67	(5)(6)(9)
(4) 50	3	19.00	(1)(8)(9)
(5) 100	3	24.67	(1)(2)(3)(7)(8)(9)
(6) 200	3	22.67	(1)(2)(3)(7)(8)(9)
(7) 300	3	14.00	(5)(6)(9)
(8) 500	3	7.33	(4)(5)(6)
(9) 750	3	2.67	(2)(3)(4)(5)(6)(7)

Jonckheere-Terpstra trend test

Test statistic	144.000
Standard Error	23.812
z statistic	-0.756
P-value (two-sided)	0.44969

Data: relative cell viability [%] for A375 cells at 12h
Factor: concentration of *Cornus officinalis* [µg/mL]

Data	survival%
Factor	conc.
Filter	time="12 h"

Sample size	27

Descriptive statistics

Factor	n	Minimum	25th percentile	Median	75th percentile	Maximum
2.5	3	89.7121	91.771	97.947	98.353	98.489
10	3	98.3396	98.392	98.549	105.179	107.389
25	3	105.2903	106.045	108.308	110.013	110.582
50	3	102.8956	103.283	104.445	106.458	107.129
100	3	110.6668	111.282	113.126	119.283	121.335
200	3	94.4629	96.905	104.232	104.443	104.513
300	3	70.5443	74.293	85.538	87.795	88.547
500	3	64.7412	66.623	72.268	73.169	73.469
750	3	62.1652	63.280	66.625	68.777	69.494

Kruskal-Wallis test

Test statistic	23.8095
Corrected for ties	23.8095
Degrees of Freedom (DF)	8
Significance level	P = 0.002466

Post-hoc analysis (Conover)

Factor	n	Average Rank	Different (P<0.05) from factor nr
(1) 2.5	3	12.00	(3)(4)(5)(8)(9)
(2) 10	3	16.67	(3)(5)(7)(8)(9)
(3) 25	3	22.33	(1)(2)(6)(7)(8)(9)
(4) 50	3	18.33	(1)(5)(7)(8)(9)
(5) 100	3	26.00	(1)(2)(4)(6)(7)(8)(9)
(6) 200	3	15.67	(3)(5)(7)(8)(9)
(7) 300	3	7.33	(2)(3)(4)(5)(6)
(8) 500	3	5.00	(1)(2)(3)(4)(5)(6)
(9) 750	3	2.67	(1)(2)(3)(4)(5)(6)

Jonckheere-Terpstra trend test

Test statistic	95.000
Standard Error	23.812
z statistic	-2.814
P-value (two-sided)	0.00490

Data: relative cell viability [%] for A375 cells at 24h
Factor: concentration of *Cornus officinalis* [µg/mL]

Data	survival%
Factor	conc.
Filter	time="24 h"

Sample size	27

Descriptive statistics

Factor	n	Minimum	25th percentile	Median	75th percentile	Maximum
2.5	3	99.6105	100.134	101.703	102.090	102.219
10	3	99.6186	99.764	100.201	103.720	104.893
25	3	100.4458	100.692	101.432	102.180	102.430
50	3	97.7106	97.989	98.825	101.165	101.945
100	3	99.0442	99.082	99.197	100.175	100.501
200	3	81.7874	84.284	91.773	93.401	93.943
300	3	53.4955	55.860	62.955	63.714	63.967
500	3	36.5614	37.236	39.260	39.796	39.975
750	3	34.9126	34.991	35.228	35.750	35.925

Kruskal-Wallis test

Test statistic	22.7513
Corrected for ties	22.7513
Degrees of Freedom (DF)	8
Significance level	P = 0.003699

Post-hoc analysis (Conover)

Factor	n	Average Rank	Different (P<0.05) from factor nr
(1) 2.5	3	21.67	(6)(7)(8)(9)
(2) 10	3	21.33	(6)(7)(8)(9)
(3) 25	3	22.67	(6)(7)(8)(9)
(4) 50	3	17.00	(6)(7)(8)(9)
(5) 100	3	17.33	(6)(7)(8)(9)
(6) 200	3	11.00	(1)(2)(3)(4)(5)(8)(9)
(7) 300	3	8.00	(1)(2)(3)(4)(5)(9)
(8) 500	3	5.00	(1)(2)(3)(4)(5)(6)
(9) 750	3	2.00	(1)(2)(3)(4)(5)(6)(7)

Jonckheere-Terpstra trend test

Test statistic	32.000
Standard Error	23.812
z statistic	-5.459
P-value (two-sided)	<0.00001

Data: relative cell viability [%] for A375 cells at 48h
Factor: concentration of *Cornus officinalis* [µg/mL]

Data	survival%
Factor	conc.
Filter	time="48 h"

Sample size	27

Descriptive statistics

Factor	n	Minimum	25th percentile	Median	75th percentile	Maximum
2.5	3	95.5130	95.888	97.012	101.926	103.565
10	3	94.1873	94.642	96.006	101.158	102.875
25	3	91.6573	91.971	92.911	100.342	102.819
50	3	88.3151	89.117	91.521	99.052	101.562
100	3	84.7648	85.676	88.410	95.444	97.789
200	3	71.7782	72.208	73.499	86.909	91.379
300	3	36.1483	37.379	41.072	59.424	65.541
500	3	15.5200	15.810	16.680	18.782	19.483
750	3	16.0219	16.727	18.842	19.334	19.498

Kruskal-Wallis test

Test statistic	21.9471
Corrected for ties	21.9471
Degrees of Freedom (DF)	8
Significance level	P = 0.005015

Post-hoc analysis (Conover)

Factor	n	Average Rank	Different (P<0.05) from factor nr
(1) 2.5	3	23.00	(5)(6)(7)(8)(9)
(2) 10	3	22.00	(6)(7)(8)(9)
(3) 25	3	20.00	(6)(7)(8)(9)
(4) 50	3	17.67	(7)(8)(9)
(5) 100	3	16.33	(1)(7)(8)(9)
(6) 200	3	12.00	(1)(2)(3)(8)(9)
(7) 300	3	8.00	(1)(2)(3)(4)(5)
(8) 500	3	3.00	(1)(2)(3)(4)(5)(6)
(9) 750	3	4.00	(1)(2)(3)(4)(5)(6)

Jonckheere-Terpstra trend test

Test statistic	31.000
Standard Error	23.812
z statistic	-5.501
P-value (two-sided)	<0.00001

Data: relative cell viability [%] for A375 cells at 72h
Factor: concentration of *Cornus officinalis* [µg/mL]

Data	survival%
Factor	conc.
Filter	time="72 h"

Sample size	27

Descriptive statistics

Factor	n	Minimum	25th percentile	Median	75th percentile	Maximum
2.5	3	98.1879	99.247	102.424	103.376	103.694
10	3	98.0030	98.410	99.631	104.237	105.772
25	3	99.2100	99.635	100.911	103.962	104.979
50	3	97.4958	97.583	97.846	104.673	106.948
100	3	94.8105	94.855	94.989	100.943	102.928
200	3	78.5594	80.680	87.040	91.756	93.328
300	3	45.5197	49.491	61.406	66.104	67.670
500	3	13.2609	13.303	13.430	14.210	14.470
750	3	14.8727	15.080	15.701	18.667	19.656

Kruskal-Wallis test

Test statistic	22.2434
Corrected for ties	22.2434
Degrees of Freedom (DF)	8
Significance level	P = 0.004484

Post-hoc analysis (Conover)

Factor	n	Average Rank	Different (P<0.05) from factor nr
(1) 2.5	3	21.33	(6)(7)(8)(9)
(2) 10	3	21.00	(6)(7)(8)(9)
(3) 25	3	21.67	(6)(7)(8)(9)
(4) 50	3	19.33	(6)(7)(8)(9)
(5) 100	3	16.67	(7)(8)(9)
(6) 200	3	11.00	(1)(2)(3)(4)(8)
(7) 300	3	8.00	(1)(2)(3)(4)(5)
(8) 500	3	2.00	(1)(2)(3)(4)(5)(6)
(9) 750	3	5.00	(1)(2)(3)(4)(5)

Jonckheere-Terpstra trend test

Test statistic	40.000
Standard Error	23.812
z statistic	-5.124
P-value (two-sided)	<0.00001
