# Supplementary material for: Time-Resolved Metabolomics Reveals Distinct, Cell- and Variety-Dependent Profiles of Prostanoids in Melanoma Cells Exposed to Fruit Extracts from Cornus mas and C. officinalis: A Pilot Study
Source: Int J Mol Sci. 2026 Aug 3;27(15):6982. doi: 10.3390/ijms27156982 (PMC13467092; doi:10.3390/ijms27156982)
Supplement: Supplementary file 1 [file ijms-27-06982-s001.zip › Figure S2.pdf]

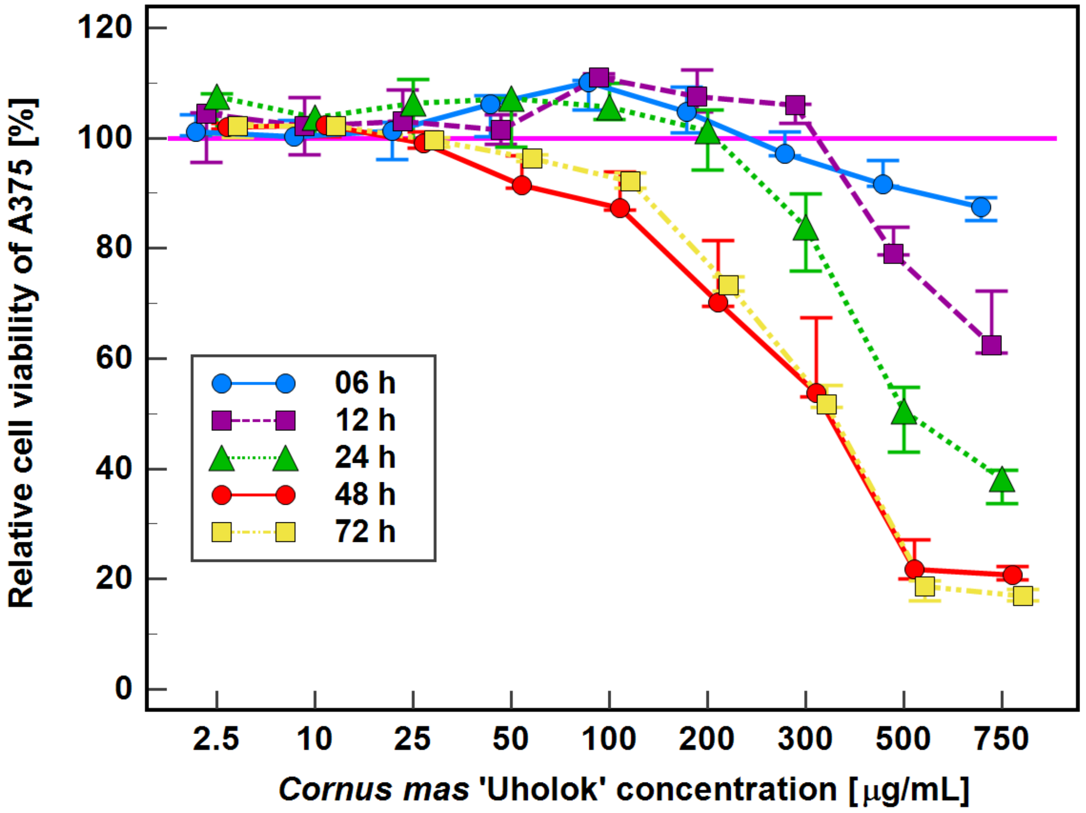

Figure S2. Viability of A375 cells treated for 6-72h with varying concentrations of Cornus mas 'Uholok' concentrations (2.5-750 µg/mL) relative to untreated cells assessed using SRB assay and expressed in [%]

Data presented as medians (circles, squares, and triangles) with interquartile range (whiskers) and analyzed using Kruskal-Wallis H test with Conover post-hoc test and Jonckheere-Terpstra trend test. SRB assay methodology as described in [58].

Accompanying statistics:

Data: relative cell viability [%] for A375 cells at 6h
Factor: concentration of *Cornus mas 'Uholok'* [µg/mL]

Kruskal-Wallis test

Data	survival%
Factor	conc.
Filter	time="06 h"

Sample size	27

Descriptive statistics

Factor	n	Minimum	25th percentile	Median	75th percentile	Maximum
2.5	3	100.2300	100.450	101.110	104.315	105.383
10	3	100.3008	100.316	100.360	103.207	104.156
25	3	94.3727	96.093	101.252	102.916	103.471
50	3	98.2152	100.223	106.246	107.804	108.324
100	3	103.4686	105.127	110.104	110.465	110.585
200	3	99.6458	100.941	104.825	109.220	110.685
300	3	96.7782	96.883	97.196	101.226	102.569
500	3	91.1544	91.272	91.624	95.980	97.431
750	3	84.2933	85.076	87.424	89.248	89.856

Kruskal-Wallis test

Test statistic	17.4074
Corrected for ties	17.4074
Degrees of Freedom (DF)	8
Significance level	P = 0.026136

Post-hoc analysis (Conover)

Factor	n	Average Rank	Different (P<0.05) from factor nr
(1) 2.5	3	16.33	(8)(9)
(2) 10	3	15.67	(8)(9)
(3) 25	3	13.67	(9)
(4) 50	3	19.00	(8)(9)
(5) 100	3	23.00	(7)(8)(9)
(6) 200	3	19.67	(8)(9)
(7) 300	3	10.67	(5)
(8) 500	3	6.00	(1)(2)(4)(5)(6)
(9) 750	3	2.00	(1)(2)(3)(4)(5)(6)

Jonckheere-Terpstra trend test

Test statistic	102.000
Standard Error	23.812
z statistic	-2.520
P-value (two-sided)	0.01174

Data: relative cell viability [%] for A375 cells at 12h
Factor: concentration of *Cornus mas 'Uholok'* [µg/mL]

Kruskal-Wallis test

Data	survival%
Factor	conc.
Filter	time="12 h"

Sample size	27

Descriptive statistics

Factor	n	Minimum	25th percentile	Median	75th percentile	Maximum
2.5	3	92.7249	95.662	104.474	104.693	104.766
10	3	95.3085	97.024	102.171	107.396	109.138
25	3	102.1606	102.366	102.983	108.831	110.780
50	3	98.0464	98.893	101.431	104.351	105.325
100	3	111.0110	111.026	111.072	111.779	112.014
200	3	103.8627	104.776	107.515	112.383	114.005
300	3	101.6950	102.765	105.975	106.098	106.140
500	3	78.7381	78.820	79.064	83.841	85.433
750	3	60.5732	61.040	62.442	72.351	75.654

Kruskal-Wallis test

Test statistic	19.5661
Corrected for ties	19.5661
Degrees of Freedom (DF)	8
Significance level	P = 0.012108

Post-hoc analysis (Conover)

Factor	n	Average Rank	Different (P<0.05) from factor nr
(1) 2.5	3	13.33	(5)(8)(9)
(2) 10	3	14.33	(5)(8)(9)
(3) 25	3	16.33	(5)(8)(9)
(4) 50	3	12.33	(5)(6)(9)
(5) 100	3	25.00	(1)(2)(3)(4)(7)(8)(9)
(6) 200	3	21.00	(4)(8)(9)
(7) 300	3	16.67	(5)(8)(9)
(8) 500	3	5.00	(1)(2)(3)(5)(6)(7)
(9) 750	3	2.00	(1)(2)(3)(4)(5)(6)(7)

Jonckheere-Terpstra trend test

Test statistic	121.000
Standard Error	23.812
z statistic	-1.722
P-value (two-sided)	0.08510

Data: relative cell viability [%] for A375 cells at 24h
Factor: concentration of *Cornus mas ‘Uholok’* [µg/mL]

Kruskal-Wallis test

Data	survival%
Factor	conc.
Filter	time="24 h"

Sample size	27

Descriptive statistics

Factor	n	Minimum	25th percentile	Median	75th percentile	Maximum
2.5	3	98.4636	100.721	107.491	108.084	108.282
10	3	99.6372	100.671	103.773	103.814	103.828
25	3	97.5561	99.766	106.397	110.609	112.013
50	3	95.5040	98.429	107.204	107.252	107.268
100	3	102.7413	103.465	105.637	109.974	111.420
200	3	91.7991	94.196	101.385	105.114	106.358
300	3	73.3484	75.994	83.931	89.898	91.887
500	3	40.5688	43.047	50.480	54.882	56.350
750	3	32.2089	33.728	38.286	39.848	40.368

Kruskal-Wallis test

Test statistic	19.3016
Corrected for ties	19.3016
Degrees of Freedom (DF)	8
Significance level	P = 0.013327

Post-hoc analysis (Conover)

Factor	n	Average Rank	Different (P<0.05) from factor nr
(1) 2.5	3	20.67	(7)(8)(9)
(2) 10	3	16.33	(8)(9)
(3) 25	3	20.00	(7)(8)(9)
(4) 50	3	18.67	(7)(8)(9)
(5) 100	3	20.33	(7)(8)(9)
(6) 200	3	14.67	(8)(9)
(7) 300	3	8.33	(1)(3)(4)(5)
(8) 500	3	5.00	(1)(2)(3)(4)(5)(6)
(9) 750	3	2.00	(1)(2)(3)(4)(5)(6)

Jonckheere-Terpstra trend test

Test statistic	59.000
Standard Error	23.812
z statistic	-4.326
P-value (two-sided)	0.00002

Data: relative cell viability [%] for A375 cells at 48h
Factor: concentration of *Cornus mas ‘Uholok’* [µg/mL]

Kruskal-Wallis test

Data	survival%
Factor	conc.
Filter	time="48 h"

Sample size	27

Descriptive statistics

Factor	n	Minimum	25th percentile	Median	75th percentile	Maximum
2.5	3	101.4958	101.613	101.963	102.758	103.022
10	3	99.4696	100.203	102.404	103.312	103.615
25	3	97.8369	98.154	99.104	101.210	101.912
50	3	90.8164	90.984	91.486	96.840	98.625
100	3	86.9260	87.024	87.316	93.872	96.058
200	3	69.3383	69.534	70.122	81.385	85.139
300	3	52.8786	53.096	53.748	67.379	71.922
500	3	19.5444	20.112	21.813	27.145	28.922
750	3	19.6231	19.885	20.669	22.286	22.825

Kruskal-Wallis test

Test statistic	24.6138
Corrected for ties	24.6138
Degrees of Freedom (DF)	8
Significance level	P = 0.001807

Post-hoc analysis (Conover)

Factor	n	Average Rank	Different (P<0.05) from factor nr
(1) 2.5	3	24.00	(4)(5)(6)(7)(8)(9)
(2) 10	3	24.33	(3)(4)(5)(6)(7)(8)(9)
(3) 25	3	20.33	(2)(5)(6)(7)(8)(9)
(4) 50	3	16.67	(1)(2)(6)(7)(8)(9)
(5) 100	3	14.67	(1)(2)(3)(6)(7)(8)(9)
(6) 200	3	10.33	(1)(2)(3)(4)(5)(8)(9)
(7) 300	3	8.67	(1)(2)(3)(4)(5)(8)(9)
(8) 500	3	3.67	(1)(2)(3)(4)(5)(6)(7)
(9) 750	3	3.33	(1)(2)(3)(4)(5)(6)(7)

Jonckheere-Terpstra trend test

Test statistic	16.000
Standard Error	23.812
z statistic	-6.131
P-value (two-sided)	<0.00001

Data: relative cell viability [%] for A375 cells at 72h
Factor: concentration of *Cornus mas ‘Uholok’* [µg/mL]

Kruskal-Wallis test

Data	survival%
Factor	conc.
Filter	time="72 h"

Sample size	27

Descriptive statistics

Factor	n	Minimum	25th percentile	Median	75th percentile	Maximum
2.5	3	99.4304	100.140	102.269	102.831	103.018
10	3	101.0691	101.364	102.247	102.599	102.717
25	3	98.0064	98.425	99.679	100.122	100.270
50	3	95.6684	95.833	96.328	97.036	97.271
100	3	90.4948	90.932	92.244	93.701	94.186
200	3	71.9245	72.264	73.281	74.919	75.465
300	3	50.9590	51.157	51.751	55.086	56.198
500	3	15.3302	16.155	18.627	19.759	20.136
750	3	15.8003	16.105	17.020	18.072	18.422

Kruskal-Wallis test

Test statistic	25.0582
Corrected for ties	25.0582
Degrees of Freedom (DF)	8
Significance level	P = 0.001520

Post-hoc analysis (Conover)

Factor	n	Average Rank	Different (P<0.05) from factor nr
(1) 2.5	3	24.00	(3)(4)(5)(6)(7)(8)(9)
(2) 10	3	24.33	(3)(4)(5)(6)(7)(8)(9)
(3) 25	3	20.67	(1)(2)(4)(5)(6)(7)(8)(9)
(4) 50	3	17.00	(1)(2)(3)(6)(7)(8)(9)
(5) 100	3	14.00	(1)(2)(3)(7)(8)(9)
(6) 200	3	11.00	(1)(2)(3)(4)(8)(9)
(7) 300	3	8.00	(1)(2)(3)(4)(5)(8)(9)
(8) 500	3	4.00	(1)(2)(3)(4)(5)(6)(7)
(9) 750	3	3.00	(1)(2)(3)(4)(5)(6)(7)

Jonckheere-Terpstra trend test

Test statistic	9.000
Standard Error	23.812
z statistic	-6.425
P-value (two-sided)	<0.00001
