# Supplementary material for: Time-Resolved Metabolomics Reveals Distinct, Cell- and Variety-Dependent Profiles of Prostanoids in Melanoma Cells Exposed to Fruit Extracts from Cornus mas and C. officinalis: A Pilot Study
Source: Int J Mol Sci. 2026 Aug 3;27(15):6982. doi: 10.3390/ijms27156982 (PMC13467092; doi:10.3390/ijms27156982)
Supplement: Supplementary file 1 [file ijms-27-06982-s001.zip › Figure S3.pdf]

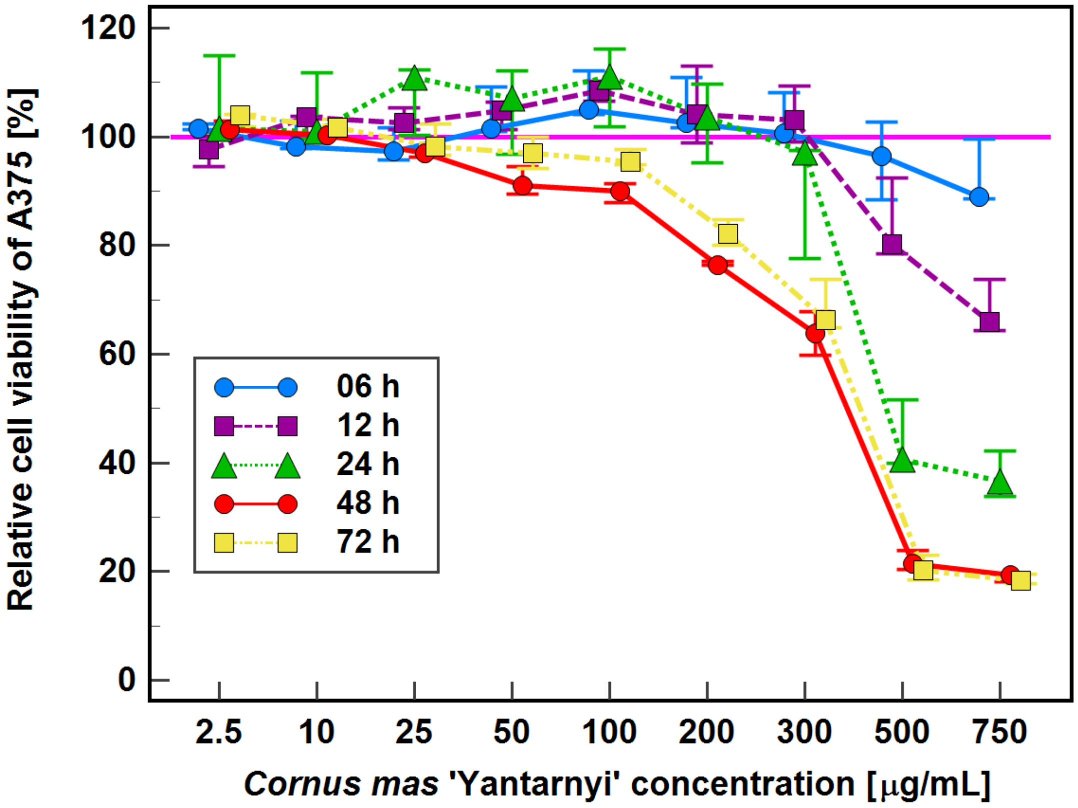

Figure S3. Viability of A375 cells treated for 6-72h with varying concentrations of Cornus mas 'Yantarnyi' concentrations (2.5-750 µg/mL) relative to untreated cells assessed using SRB assay and expressed in [%]

Data presented as medians (circles, squares, and triangles) with interquartile range (whiskers) and analyzed using Kruskal-Wallis H test with Conover post-hoc test and Jonckheere-Terpstra trend test. SRB assay methodology as described in [58].

Accompanying statistics:

Data: relative cell viability [%] for A375 cells at 6h
Factor: concentration of C. 'Yantarnyi' [µg/mL]

Kruskal-Wallis test

Data	survival%
Factor	conc.
Filter	time="06 h"

Sample size	27

Descriptive statistics

Factor	n	Minimum	25th percentile	Median	75th percentile	Maximum
2.5	3	101.1971	101.275	101.510	102.302	102.566
10	3	97.5928	97.764	98.277	102.923	104.472
25	3	95.1485	95.687	97.301	101.740	103.220
50	3	100.7834	100.968	101.521	109.244	111.818
100	3	103.7175	104.049	105.045	112.234	114.630
200	3	101.5048	101.772	102.575	111.009	113.820
300	3	99.4799	99.766	100.625	108.186	110.706
500	3	85.6524	88.374	96.539	102.723	104.784
750	3	88.4688	88.586	88.938	99.517	103.044

Kruskal-Wallis test

Test statistic	9.6508
Corrected for ties	9.6508
Degrees of Freedom (DF)	8
Significance level	P = 0.290396

Factor	n	Average Rank
(1) 2.5	3	14.00
(2) 10	3	12.00
(3) 25	3	9.67
(4) 50	3	17.00
(5) 100	3	23.33
(6) 200	3	18.67
(7) 300	3	14.33
(8) 500	3	9.33
(9) 750	3	7.67

Jonckheere-Terpstra trend test

Test statistic	146.000
Standard Error	23.812
z statistic	-0.672
P-value (two-sided)	0.50162

Data: relative cell viability [%] for A375 cells at 12h
Factor: concentration of C. mas 'Yantarnyi' [µg/mL]

Kruskal-Wallis test

Data	survival%
Factor	conc.
Filter	time="12 h"

Sample size	27

Descriptive statistics

Factor	n	Minimum	25th percentile	Median	75th percentile	Maximum
2.5	3	93.4857	94.549	97.739	100.125	100.920
10	3	99.2153	100.292	103.520	103.857	103.970
25	3	100.8788	101.286	102.509	105.291	106.219
50	3	100.2333	101.373	104.792	106.383	106.913
100	3	105.9283	106.548	108.408	110.226	110.832
200	3	97.1417	98.871	104.060	113.058	116.057
300	3	97.6564	99.029	103.148	109.311	111.365
500	3	77.9068	78.506	80.305	92.469	96.524
750	3	63.9049	64.416	65.950	73.836	76.465

Kruskal-Wallis test

Test statistic	17.8519
Corrected for ties	17.8519
Degrees of Freedom (DF)	8
Significance level	P = 0.022365

Post-hoc analysis (Conover)

Factor	n	Average Rank	Different (P<0.05) from factor nr
(1) 2.5	3	10.00	(5)
(2) 10	3	15.33	(8)(9)
(3) 25	3	16.67	(8)(9)
(4) 50	3	18.33	(8)(9)
(5) 100	3	23.33	(1)(8)(9)
(6) 200	3	18.00	(8)(9)
(7) 300	3	17.00	(8)(9)
(8) 500	3	5.33	(2)(3)(4)(5)(6)(7)
(9) 750	3	2.00	(2)(3)(4)(5)(6)(7)

Jonckheere-Terpstra trend test

Test statistic	128.000
Standard Error	23.812
z statistic	-1.428
P-value (two-sided)	0.15333

Data: relative cell viability [%] for A375 cells at 24h
Factor: concentration of C. mas ‘Yantarnyi’ [µg/mL]

Kruskal-Wallis test

Data	survival%
Factor	conc.
Filter	time="24 h"

Sample size	27

Descriptive statistics

Factor	n	Minimum	25th percentile	Median	75th percentile	Maximum
2.5	3	100.6624	100.857	101.442	114.890	119.372
10	3	99.1537	99.628	101.051	111.873	115.480
25	3	96.8606	100.373	110.908	112.277	112.733
50	3	93.3151	96.781	107.178	112.080	113.714
100	3	98.7373	101.842	111.155	116.203	117.886
200	3	92.4371	95.249	103.683	109.792	111.828
300	3	71.2301	77.703	97.123	97.531	97.667
500	3	39.7271	39.950	40.618	51.666	55.348
750	3	32.9158	33.841	36.615	42.261	44.144

Kruskal-Wallis test

Test statistic	16.8254
Corrected for ties	16.8254
Degrees of Freedom (DF)	8
Significance level	P = 0.031979

Post-hoc analysis (Conover)

Factor	n	Average Rank	Different (P<0.05) from factor nr
(1) 2.5	3	19.67	(8)(9)
(2) 10	3	18.33	(8)(9)
(3) 25	3	17.67	(8)(9)
(4) 50	3	17.33	(8)(9)
(5) 100	3	20.00	(7)(8)(9)
(6) 200	3	16.00	(8)(9)
(7) 300	3	10.00	(5)
(8) 500	3	4.33	(1)(2)(3)(4)(5)(6)
(9) 750	3	2.67	(1)(2)(3)(4)(5)(6)

Jonckheere-Terpstra trend test

Test statistic	69.000
Standard Error	23.812
z statistic	-3.906
P-value (two-sided)	0.00009

Data: relative cell viability [%] for A375 cells at 48h
Factor: concentration of C. mas ‘Yantarnyi’ [µg/mL]

Kruskal-Wallis test

Data	survival%
Factor	conc.
Filter	time="48 h"

Sample size	27

Descriptive statistics

Factor	n	Minimum	25th percentile	Median	75th percentile	Maximum
2.5	3	99.2957	99.807	101.342	102.950	103.486
10	3	99.4696	99.695	100.373	100.640	100.729
25	3	96.0601	96.285	96.959	98.103	98.485
50	3	88.9419	89.483	91.106	94.546	95.693
100	3	87.1921	87.907	90.052	91.399	91.848
200	3	76.2913	76.336	76.472	77.044	77.235
300	3	58.4412	59.777	63.785	67.913	69.289
500	3	20.0945	20.439	21.474	23.932	24.751
750	3	17.7583	18.166	19.389	19.481	19.511

Kruskal-Wallis test

Test statistic	25.3333
Corrected for ties	25.3333
Degrees of Freedom (DF)	8
Significance level	P = 0.001365

Post-hoc analysis (Conover)

Factor	n	Average Rank	Different (P<0.05) from factor nr
(1) 2.5	3	25.00	(3)(4)(5)(6)(7)(8)(9)
(2) 10	3	24.00	(3)(4)(5)(6)(7)(8)(9)
(3) 25	3	20.00	(1)(2)(4)(5)(6)(7)(8)(9)
(4) 50	3	16.00	(1)(2)(3)(6)(7)(8)(9)
(5) 100	3	15.00	(1)(2)(3)(6)(7)(8)(9)
(6) 200	3	11.00	(1)(2)(3)(4)(5)(7)(8)(9)
(7) 300	3	8.00	(1)(2)(3)(4)(5)(6)(8)(9)
(8) 500	3	5.00	(1)(2)(3)(4)(5)(6)(7)(9)
(9) 750	3	2.00	(1)(2)(3)(4)(5)(6)(7)(8)

Jonckheere-Terpstra trend test

Test statistic	6.000
Standard Error	23.812
z statistic	-6.551
P-value (two-sided)	<0.00001

Data: relative cell viability [%] for A375 cells at 72h
Factor: concentration of C. mas ‘Yantarnyi’ [µg/mL]

Kruskal-Wallis test

Data	survival%
Factor	conc.
Filter	time="72 h"

Sample size	27

Descriptive statistics

Factor	n	Minimum	25th percentile	Median	75th percentile	Maximum
2.5	3	101.1126	101.807	103.890	104.163	104.254
10	3	101.1004	101.233	101.630	103.322	103.886
25	3	96.0144	96.576	98.260	102.403	103.784
50	3	93.2109	94.149	96.962	99.930	100.919
100	3	94.5854	94.809	95.480	97.679	98.413
200	3	79.2437	79.973	82.163	84.731	85.587
300	3	64.3451	64.836	66.309	73.812	76.313
500	3	17.8167	18.402	20.156	23.005	23.954
750	3	17.5621	17.749	18.310	19.462	19.846

Kruskal-Wallis test

Test statistic	24.2116
Corrected for ties	24.2116
Degrees of Freedom (DF)	8
Significance level	P = 0.002112

Post-hoc analysis (Conover)

Factor	n	Average Rank	Different (P<0.05) from factor nr
(1) 2.5	3	25.00	(3)(4)(5)(6)(7)(8)(9)
(2) 10	3	23.00	(4)(5)(6)(7)(8)(9)
(3) 25	3	19.33	(1)(6)(7)(8)(9)
(4) 50	3	16.67	(1)(2)(6)(7)(8)(9)
(5) 100	3	16.00	(1)(2)(6)(7)(8)(9)
(6) 200	3	11.00	(1)(2)(3)(4)(5)(8)(9)
(7) 300	3	8.00	(1)(2)(3)(4)(5)(9)
(8) 500	3	4.33	(1)(2)(3)(4)(5)(6)
(9) 750	3	2.67	(1)(2)(3)(4)(5)(6)(7)

Jonckheere-Terpstra trend test

Test statistic	16.000
Standard Error	23.812
z statistic	-6.131
P-value (two-sided)	<0.00001
