# Supplementary material for: Time-Resolved Metabolomics Reveals Distinct, Cell- and Variety-Dependent Profiles of Prostanoids in Melanoma Cells Exposed to Fruit Extracts from Cornus mas and C. officinalis: A Pilot Study
Source: Int J Mol Sci. 2026 Aug 3;27(15):6982. doi: 10.3390/ijms27156982 (PMC13467092; doi:10.3390/ijms27156982)
Supplement: Supplementary file 1 [file ijms-27-06982-s001.zip › Figure S4.pdf]

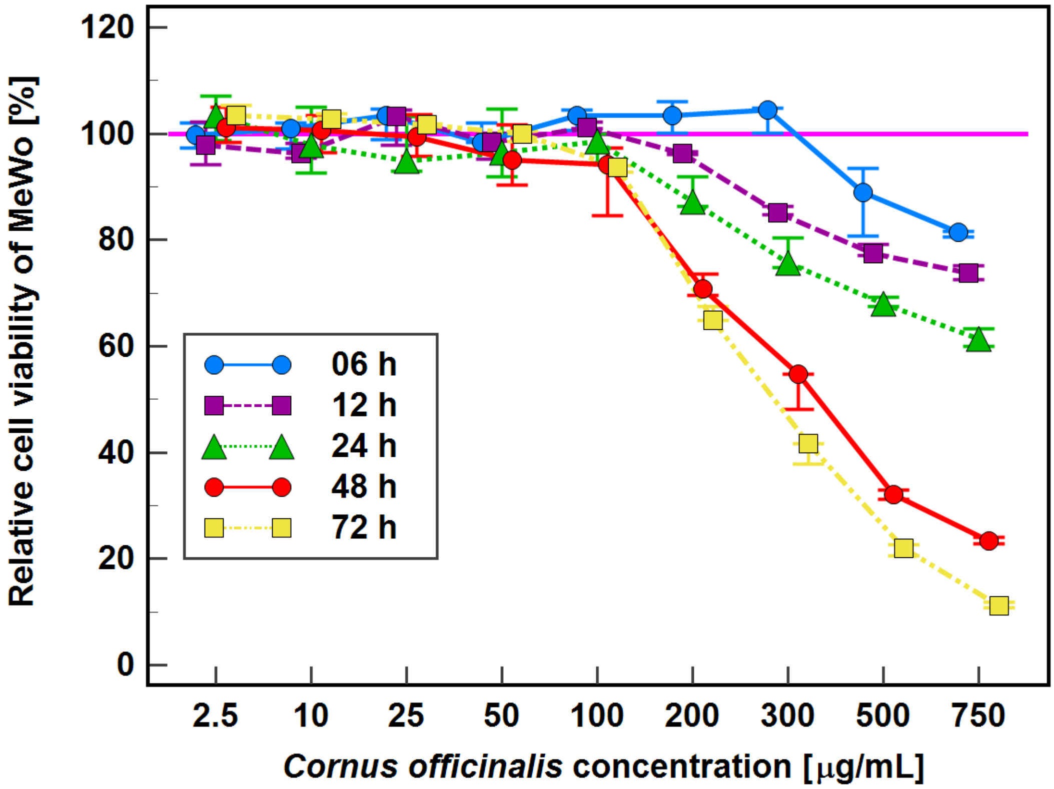

Figure S4. Viability of Mewo cells treated for 6-72h with varying concentrations of *Cornus officinalis* concentrations (2.5-750 µg/mL) relative to untreated cells assessed using SRB assay and expressed in [%]

Data presented as medians (circles, squares, and triangles) with interquartile range (whiskers) and analyzed using Kruskal-Wallis H test with Conover post-hoc test and Jonckheere-Terpstra trend test. SRB assay methodology as described in [58].

Accompanying statistics:

Data: relative cell viability [%] for Mewo cells at 6h
Factor: concentration of *Cornus officinalis* [µg/mL]

Kruskal-Wallis test

Data	survival%
Factor	conc.
Filter	time="06 h"
Sample size	27

Descriptive statistics

Factor	n	Minimum	25th percentile	Median	75th percentile	Maximum
2.5	3	96.6010	97.400	99.796	102.096	102.863
10	3	95.8010	97.092	100.963	101.975	102.312
25	3	97.4888	98.963	103.385	104.620	105.032
50	3	98.2990	98.330	98.422	101.986	103.174
100	3	99.7331	100.645	103.380	104.406	104.748
200	3	98.9711	100.094	103.463	105.985	106.826
300	3	98.6773	100.121	104.451	104.741	104.838
500	3	77.9362	80.684	88.928	93.501	95.025
750	3	80.2513	80.557	81.475	81.668	81.733

Kruskal-Wallis test

Test statistic	16.7937
Corrected for ties	16.7937
Degrees of Freedom (DF)	8
Significance level	P = 0.032331

Post-hoc analysis (Conover)

Factor	n	Average Rank	Different (P<0.05) from factor nr
(1) 2.5	3	13.67	(9)
(2) 10	3	13.33	(9)
(3) 25	3	18.67	(8)(9)
(4) 50	3	13.33	(9)
(5) 100	3	19.33	(8)(9)
(6) 200	3	20.67	(8)(9)
(7) 300	3	20.00	(8)(9)
(8) 500	3	4.00	(3)(5)(6)(7)
(9) 750	3	3.00	(1)(2)(3)(4)(5)(6)(7)

Jonckheere-Terpstra trend test

Test statistic	129.000
Standard Error	23.812
z statistic	-1.386
P-value (two-sided)	0.16579

Data: relative cell viability [%] for Mewo cells at 12h
Factor: concentration of *Cornus officinalis* [µg/mL]

Kruskal-Wallis test

Data	survival%
Factor	conc.
Filter	time="12 h"
Sample size	27

Descriptive statistics

Factor	n	Minimum	25th percentile	Median	75th percentile	Maximum
2.5	3	93.0077	94.207	97.806	102.216	103.686
10	3	95.1957	95.485	96.355	98.242	98.871
25	3	96.0031	97.836	103.336	104.548	104.952
50	3	94.1684	95.237	98.442	100.080	100.626
100	3	100.9762	101.041	101.235	102.247	102.585
200	3	96.1777	96.185	96.208	96.551	96.665
300	3	84.5404	84.694	85.153	86.285	86.662
500	3	77.0909	77.160	77.369	79.190	79.797
750	3	72.1790	72.582	73.789	75.143	75.594

Kruskal-Wallis test

Test statistic	20.5503
Corrected for ties	20.5503
Degrees of Freedom (DF)	8
Significance level	P = 0.008443

Post-hoc analysis (Conover)

Factor	n	Average Rank	Different (P<0.05) from factor nr
(1) 2.5	3	18.00	(7)(8)(9)
(2) 10	3	16.00	(7)(8)(9)
(3) 25	3	21.67	(7)(8)(9)
(4) 50	3	17.00	(7)(8)(9)
(5) 100	3	23.00	(6)(7)(8)(9)
(6) 200	3	15.33	(5)(8)(9)
(7) 300	3	8.00	(1)(2)(3)(4)(5)
(8) 500	3	5.00	(1)(2)(3)(4)(5)(6)
(9) 750	3	2.00	(1)(2)(3)(4)(5)(6)

Jonckheere-Terpstra trend test

Test statistic	69.000
Standard Error	23.812
z statistic	-3.906
P-value (two-sided)	0.00009

Data: relative cell viability [%] for Mewo cells at 24h
Factor: concentration of *Cornus officinalis* [µg/mL]

Kruskal-Wallis test

Data	survival%
Factor	conc.
Filter	time="24 h"

Sample size	27

Descriptive statistics

Factor	n	Minimum	25th percentile	Median	75th percentile	Maximum
2.5	3	97.1420	98.697	103.361	107.159	108.424
10	3	90.8206	92.578	97.851	105.032	107.426
25	3	92.2543	92.923	94.931	103.174	105.922
50	3	90.5110	91.987	96.416	104.716	107.483
100	3	93.1998	94.516	98.463	99.939	100.432
200	3	85.9693	86.282	87.222	91.844	93.385
300	3	74.4827	74.790	75.710	80.423	81.994
500	3	67.2398	67.432	68.008	69.278	69.702
750	3	59.5233	59.979	61.347	63.351	64.019

Kruskal-Wallis test

Test statistic	21.0582
Corrected for ties	21.0582
Degrees of Freedom (DF)	8
Significance level	P = 0.006994

Post-hoc analysis (Conover)

Factor	n	Average Rank	Different (P<0.05) from factor nr
(1) 2.5	3	23.00	(6)(7)(8)(9)
(2) 10	3	19.33	(7)(8)(9)
(3) 25	3	18.33	(7)(8)(9)
(4) 50	3	18.67	(7)(8)(9)
(5) 100	3	19.33	(7)(8)(9)
(6) 200	3	12.33	(1)(8)(9)
(7) 300	3	8.00	(1)(2)(3)(4)(5)
(8) 500	3	5.00	(1)(2)(3)(4)(5)(6)
(9) 750	3	2.00	(1)(2)(3)(4)(5)(6)

Jonckheere-Terpstra trend test

Test statistic	41.000
Standard Error	23.812
z statistic	-5.082
P-value (two-sided)	<0.00001

Data: relative cell viability [%] for Mewo cells at 48h
Factor: concentration of *Cornus officinalis* [µg/mL]

Kruskal-Wallis test

Data	survival%
Factor	conc.
Filter	time="48 h"

Sample size	27

Descriptive statistics

Factor	n	Minimum	25th percentile	Median	75th percentile	Maximum
2.5	3	97.5355	98.443	101.167	105.085	106.391
10	3	94.9589	96.383	100.654	103.510	104.463
25	3	94.5148	95.755	99.474	103.546	104.903
50	3	88.6995	90.301	95.107	101.749	103.963
100	3	81.3716	84.572	94.172	97.326	98.377
200	3	69.0932	69.510	70.761	73.612	74.562
300	3	45.9781	48.159	54.703	54.847	54.895
500	3	31.0289	31.274	32.008	33.010	33.344
750	3	22.6450	22.841	23.428	24.053	24.261

Kruskal-Wallis test

Test statistic	22.8360
Corrected for ties	22.8360
Degrees of Freedom (DF)	8
Significance level	P = 0.003581

Post-hoc analysis (Conover)

Factor	n	Average Rank	Different (P<0.05) from factor nr
(1) 2.5	3	23.00	(5)(6)(7)(8)(9)
(2) 10	3	21.33	(6)(7)(8)(9)
(3) 25	3	21.00	(6)(7)(8)(9)
(4) 50	3	18.67	(6)(7)(8)(9)
(5) 100	3	16.00	(1)(7)(8)(9)
(6) 200	3	11.00	(1)(2)(3)(4)(8)(9)
(7) 300	3	8.00	(1)(2)(3)(4)(5)(9)
(8) 500	3	5.00	(1)(2)(3)(4)(5)(6)
(9) 750	3	2.00	(1)(2)(3)(4)(5)(6)(7)

Jonckheere-Terpstra trend test

Test statistic	24.000
Standard Error	23.812
z statistic	-5.795
P-value (two-sided)	<0.00001

Data: relative cell viability [%] for Mewo cells at 72h
Factor: concentration of *Cornus officinalis* [µg/mL]

Kruskal-Wallis test

Data	survival%
Factor	conc.
Filter	time="72 h"

Sample size	27

Descriptive statistics

Factor	n	Minimum	25th percentile	Median	75th percentile	Maximum
2.5	3	99.3030	100.326	103.395	105.301	105.936
10	3	102.2007	102.318	102.668	103.806	104.185
25	3	100.7488	100.993	101.727	101.782	101.800
50	3	98.0249	98.495	99.906	100.439	100.617
100	3	92.3875	92.717	93.707	94.058	94.175
200	3	64.7996	64.816	64.864	67.560	68.459
300	3	36.6596	37.926	41.723	41.758	41.769
500	3	20.0559	20.548	22.026	22.603	22.795
750	3	10.8110	10.891	11.132	11.859	12.102

Kruskal-Wallis test

Test statistic	24.7725
Corrected for ties	24.7725
Degrees of Freedom (DF)	8
Significance level	P = 0.001699

Post-hoc analysis (Conover)

Factor	n	Average Rank	Different (P<0.05) from factor nr
(1) 2.5	3	23.00	(4)(5)(6)(7)(8)(9)
(2) 10	3	24.33	(4)(5)(6)(7)(8)(9)
(3) 25	3	21.00	(5)(6)(7)(8)(9)
(4) 50	3	17.67	(1)(2)(5)(6)(7)(8)(9)
(5) 100	3	14.00	(1)(2)(3)(4)(7)(8)(9)
(6) 200	3	11.00	(1)(2)(3)(4)(8)(9)
(7) 300	3	8.00	(1)(2)(3)(4)(5)(9)
(8) 500	3	5.00	(1)(2)(3)(4)(5)(6)
(9) 750	3	2.00	(1)(2)(3)(4)(5)(6)(7)

Jonckheere-Terpstra trend test

Test statistic	9.000
Standard Error	23.812
z statistic	-6.425
P-value (two-sided)	<0.00001
