# Supplementary material for: Time-Resolved Metabolomics Reveals Distinct, Cell- and Variety-Dependent Profiles of Prostanoids in Melanoma Cells Exposed to Fruit Extracts from Cornus mas and C. officinalis: A Pilot Study
Source: Int J Mol Sci. 2026 Aug 3;27(15):6982. doi: 10.3390/ijms27156982 (PMC13467092; doi:10.3390/ijms27156982)
Supplement: Supplementary file 1 [file ijms-27-06982-s001.zip › Figure S5.pdf]

Lewandowski et al.

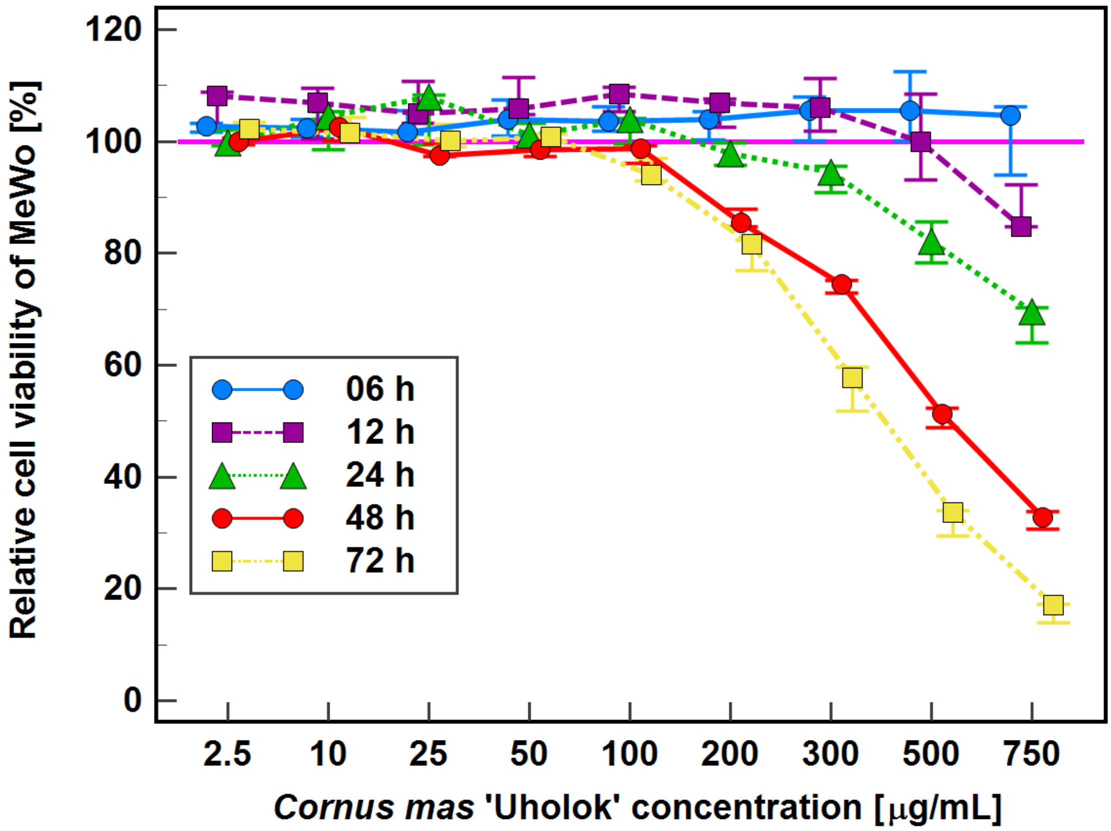

Figure S5. Viability of Mewo cells treated for 6-72h with varying concentrations of Cornus mas ‘Uholok’ concentrations (2.5-750 μg/mL) relative to untreated cells assessed using SRB assay and expressed in [%]

Data presented as medians (circles, squares, and triangles) with interquartile range (whiskers) and analyzed using Kruskal-Wallis H test with Conover post-hoc test and Jonckheere-Terpstra trend test. SRB assay methodology as described in [58].

Accompanying statistics:

Data: relative cell viability [%] for Mewo cells at 6h
Factor: concentration of *Cornus mas ‘Uholok’* [μg/mL]

Kruskal-Wallis test

Data	survival%
Factor	conc.
Filter	time="06 h"
Sample size	27

Descriptive statistics

Factor	n	Minimum	25th percentile	Median	75th percentile	Maximum
2.5	3	101.2783	101.649	102.761	103.172	103.309
10	3	100.1712	100.735	102.425	104.041	104.579
25	3	101.2003	101.343	101.772	105.443	106.667
50	3	100.0023	100.979	103.908	107.432	108.606
100	3	101.2493	101.841	103.615	106.294	107.187
200	3	99.0642	100.270	103.886	105.406	105.913
300	3	98.2886	100.115	105.594	107.961	108.751
500	3	98.2816	100.077	105.462	112.559	114.924
750	3	90.4932	94.048	104.713	106.304	106.834

Kruskal-Wallis test

Test statistic	1.3651
Corrected for ties	1.3651
Degrees of Freedom (DF)	8
Significance level	P = 0.994728

Factor	n	Average Rank
(1) 2.5	3	11.33
(2) 10	3	11.33
(3) 25	3	13.00
(4) 50	3	15.33
(5) 100	3	15.33
(6) 200	3	13.33
(7) 300	3	16.33
(8) 500	3	16.00
(9) 750	3	14.00

Jonckheere-Terpstra trend test

Test statistic	177.000
Standard Error	23.812
z statistic	0.630
P-value (two-sided)	0.52873

Data: relative cell viability [%] for Mewo cells at 12h
Factor: concentration of *Cornus mas ‘Uholok’* [μg/mL]

Kruskal-Wallis test

Data	survival%
Factor	conc.
Filter	time="12 h"
Sample size	27

Descriptive statistics

Factor	n	Minimum	25th percentile	Median	75th percentile	Maximum
2.5	3	100.4499	102.390	108.212	108.857	109.072
10	3	98.4019	100.542	106.961	109.508	110.357
25	3	102.6026	103.188	104.945	110.783	112.729
50	3	104.5552	104.865	105.796	111.454	113.340
100	3	104.2598	105.311	108.466	109.637	110.027
200	3	101.0062	102.485	106.922	107.645	107.886
300	3	100.4801	101.853	105.973	111.267	113.031
500	3	90.9378	93.169	99.863	108.542	111.435
750	3	84.6650	84.704	84.822	92.215	94.680

Kruskal-Wallis test

Test statistic	8.8254
Corrected for ties	8.8254
Degrees of Freedom (DF)	8
Significance level	P = 0.357239

Factor	n	Average Rank
(1) 2.5	3	15.67
(2) 10	3	15.00
(3) 25	3	16.00
(4) 50	3	17.67
(5) 100	3	17.67
(6) 200	3	14.33
(7) 300	3	16.33
(8) 500	3	11.00
(9) 750	3	2.33

Jonckheere-Terpstra trend test

Test statistic	114.000
Standard Error	23.812
z statistic	-2.016
P-value (two-sided)	0.04382

Data: relative cell viability [%] for Mewo cells at 24h
Factor: concentration of *Cornus mas 'Uholok'* [µg/mL]

Kruskal-Wallis test

Data	survival%
Factor	conc.
Filter	time="24 h"

Sample size	27

Descriptive statistics

Factor	n	Minimum	25th percentile	Median	75th percentile	Maximum
2.5	3	99.1595	99.320	99.800	102.076	102.834
10	3	96.5699	98.561	104.533	105.424	105.720
25	3	96.5922	99.441	107.988	108.273	108.369
50	3	98.3428	99.040	101.133	103.226	103.924
100	3	98.0375	99.525	103.988	104.098	104.134
200	3	95.1385	95.808	97.818	99.759	100.405
300	3	89.6205	90.832	94.465	95.551	95.913
500	3	77.0276	78.315	82.179	85.702	86.877
750	3	62.0709	63.943	69.561	70.364	70.631

Kruskal-Wallis test

Test statistic	19.8413
Corrected for ties	19.8413
Degrees of Freedom (DF)	8
Significance level	P = 0.010954

Post-hoc analysis (Conover)

Factor	n	Average Rank	Different (P<0.05) from factor nr
(1) 2.5	3	17.67	(7)(8)(9)
(2) 10	3	20.00	(7)(8)(9)
(3) 25	3	21.67	(6)(7)(8)(9)
(4) 50	3	18.33	(7)(8)(9)
(5) 100	3	19.67	(7)(8)(9)
(6) 200	3	13.33	(3)(8)(9)
(7) 300	3	8.33	(1)(2)(3)(4)(5)
(8) 500	3	5.00	(1)(2)(3)(4)(5)(6)
(9) 750	3	2.00	(1)(2)(3)(4)(5)(6)

Jonckheere-Terpstra trend test

Test statistic	57.000
Standard Error	23.812
z statistic	-4.410
P-value (two-sided)	0.00001

Data: relative cell viability [%] for Mewo cells at 48h
Factor: concentration of *Cornus mas 'Uholok'* [µg/mL]

Kruskal-Wallis test

Data	survival%
Factor	conc.
Filter	time="48 h"

Sample size	27

Descriptive statistics

Factor	n	Minimum	25th percentile	Median	75th percentile	Maximum
2.5	3	99.2468	99.404	99.876	100.685	100.954
10	3	99.3626	100.179	102.630	103.040	103.177
25	3	97.2178	97.280	97.468	99.550	100.244
50	3	97.0131	97.400	98.560	100.778	101.517
100	3	95.1381	96.049	98.781	99.185	99.320
200	3	84.5930	84.807	85.447	87.959	88.796
300	3	72.3213	72.865	74.498	75.165	75.387
500	3	47.9495	48.760	51.192	52.404	52.808
750	3	30.1106	30.775	32.768	33.819	34.170

Kruskal-Wallis test

Test statistic	23.3016
Corrected for ties	23.3016
Degrees of Freedom (DF)	8
Significance level	P = 0.002998

Post-hoc analysis (Conover)

Factor	n	Average Rank	Different (P<0.05) from factor nr
(1) 2.5	3	21.67	(6)(7)(8)(9)
(2) 10	3	24.67	(3)(4)(5)(6)(7)(8)(9)
(3) 25	3	18.00	(2)(6)(7)(8)(9)
(4) 50	3	18.67	(2)(6)(7)(8)(9)
(5) 100	3	17.00	(2)(6)(7)(8)(9)
(6) 200	3	11.00	(1)(2)(3)(4)(5)(8)(9)
(7) 300	3	8.00	(1)(2)(3)(4)(5)(9)
(8) 500	3	5.00	(1)(2)(3)(4)(5)(6)
(9) 750	3	2.00	(1)(2)(3)(4)(5)(6)(7)

Jonckheere-Terpstra trend test

Test statistic	28.000
Standard Error	23.812
z statistic	-5.627
P-value (two-sided)	<0.00001

Data: relative cell viability [%] for Mewo cells at 72h
Factor: concentration of *Cornus mas 'Uholok'* [µg/mL]

Kruskal-Wallis test

Data	survival%
Factor	conc.
Filter	time="72 h"

Sample size	27

Descriptive statistics

Factor	n	Minimum	25th percentile	Median	75th percentile	Maximum
2.5	3	101.2500	101.478	102.162	103.514	103.965
10	3	101.2405	101.289	101.434	104.353	105.326
25	3	98.8415	99.140	100.037	103.025	104.020
50	3	99.6048	99.909	100.823	101.280	101.432
100	3	92.5312	92.884	93.943	96.906	97.894
200	3	75.3370	76.925	81.687	82.361	82.585
300	3	49.8595	51.811	57.666	59.581	60.219
500	3	28.2142	29.555	33.577	33.938	34.059
750	3	12.9374	13.970	17.068	17.307	17.386

Kruskal-Wallis test

Test statistic	24.2222
Corrected for ties	24.2222
Degrees of Freedom (DF)	8
Significance level	P = 0.002103

Post-hoc analysis (Conover)

Factor	n	Average Rank	Different (P<0.05) from factor nr
(1) 2.5	3	23.33	(5)(6)(7)(8)(9)
(2) 10	3	23.33	(5)(6)(7)(8)(9)
(3) 25	3	20.00	(5)(6)(7)(8)(9)
(4) 50	3	19.33	(5)(6)(7)(8)(9)
(5) 100	3	14.00	(1)(2)(3)(4)(7)(8)(9)
(6) 200	3	11.00	(1)(2)(3)(4)(8)(9)
(7) 300	3	8.00	(1)(2)(3)(4)(5)(9)
(8) 500	3	5.00	(1)(2)(3)(4)(5)(6)
(9) 750	3	2.00	(1)(2)(3)(4)(5)(6)(7)

Jonckheere-Terpstra trend test

Test statistic	16.000
Standard Error	23.812
z statistic	-6.131
P-value (two-sided)	<0.00001
