# Supplementary material for: Time-Resolved Metabolomics Reveals Distinct, Cell- and Variety-Dependent Profiles of Prostanoids in Melanoma Cells Exposed to Fruit Extracts from Cornus mas and C. officinalis: A Pilot Study
Source: Int J Mol Sci. 2026 Aug 3;27(15):6982. doi: 10.3390/ijms27156982 (PMC13467092; doi:10.3390/ijms27156982)
Supplement: Supplementary file 1 [file ijms-27-06982-s001.zip › Figure S6.pdf]

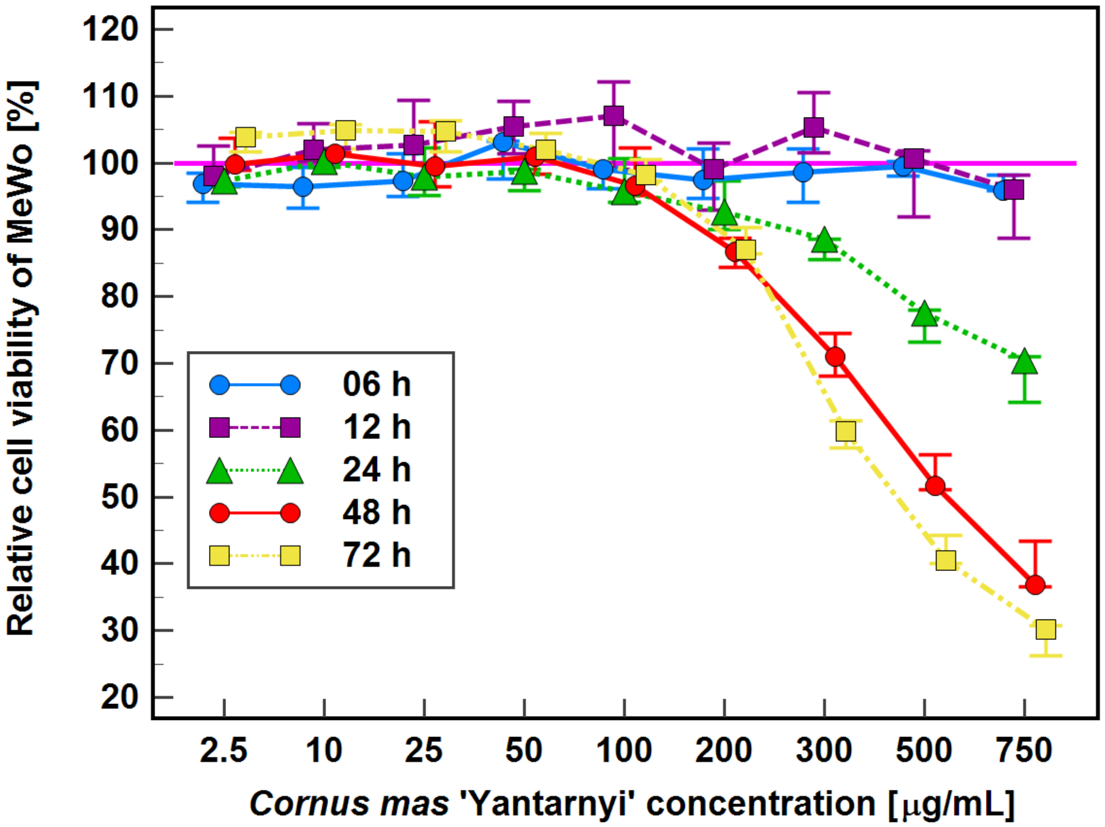

Figure S6. Viability of Mewo cells treated for 6-72h with varying concentrations of *Cornus mas 'Yantarnyi'* concentrations (2.5-750 µg/mL) relative to untreated cells assessed using SRB assay and expressed in [%]

Data presented as medians (circles, squares, and triangles) with interquartile range (whiskers) and analyzed using Kruskal-Wallis H test with Conover post-hoc test and Jonckheere-Terpstra trend test. SRB assay methodology as described in [58].

Accompanying statistics:

Data: relative cell viability [%] for Mewo cells at 6h
Factor: concentration of C. mas 'Yantarnyi' [µg/mL]

Kruskal-Wallis test

Data	survival%
Factor	conc.
Filter	time="06 h"

Sample size	27

Descriptive statistics

Factor	n	Minimum	25th percentile	Median	75th percentile	Maximum
2.5	3	93.2955	94.200	96.915	98.422	98.924
10	3	92.2461	93.294	96.438	99.330	100.294
25	3	94.2577	95.015	97.287	101.397	102.767
50	3	95.7914	97.623	103.119	103.273	103.324
100	3	95.1313	96.134	99.141	99.321	99.381
200	3	93.8285	94.747	97.502	102.070	103.593
300	3	92.6835	94.175	98.651	102.058	103.194
500	3	97.5194	98.025	99.541	100.235	100.466
750	3	95.7727	95.802	95.891	98.173	98.934

Kruskal-Wallis test

Test statistic	4.1587
Corrected for ties	4.1587
Degrees of Freedom (DF)	8
Significance level	P = 0.842526

Factor	n	Average Rank
(1) 2.5	3	10.00
(2) 10	3	10.67
(3) 25	3	13.33
(4) 50	3	19.33
(5) 100	3	14.33
(6) 200	3	14.67
(7) 300	3	14.00
(8) 500	3	18.67
(9) 750	3	11.00

Jonckheere-Terpstra trend test

Test statistic	177.000
Standard Error	23.812
z statistic	0.630
P-value (two-sided)	0.52873

Data: relative cell viability [%] for Mewo cells at 12h
Factor: concentration of C. mas 'Yantarnyi' [µg/mL]

Kruskal-Wallis test

Data	survival%
Factor	conc.
Filter	time="12 h"

Sample size	27

Descriptive statistics

Factor	n	Minimum	25th percentile	Median	75th percentile	Maximum
2.5	3	97.4196	97.587	98.089	102.515	103.990
10	3	99.7073	100.257	101.905	105.920	107.258
25	3	99.0038	99.924	102.685	109.321	111.533
50	3	99.9613	101.329	105.432	109.205	110.462
100	3	96.4563	99.096	107.014	112.146	113.856
200	3	90.9337	92.984	99.137	103.018	104.312
300	3	100.2592	101.535	105.364	110.484	112.190
500	3	89.0458	91.965	100.722	101.767	102.115
750	3	86.3067	88.736	96.022	98.231	98.967

Kruskal-Wallis test

Test statistic	10.3175
Corrected for ties	10.3175
Degrees of Freedom (DF)	8
Significance level	P = 0.243447

Factor	n	Average Rank
(1) 2.5	3	10.33
(2) 10	3	16.33
(3) 25	3	17.00
(4) 50	3	19.00
(5) 100	3	18.00
(6) 200	3	10.67
(7) 300	3	19.67
(8) 500	3	10.67
(9) 750	3	4.33

Jonckheere-Terpstra trend test

Test statistic	135.000
Standard Error	23.812
z statistic	-1.134
P-value (two-sided)	0.25684

Data: relative cell viability [%] for Mewo cells at 24h
Factor: concentration of C. mas 'Yantarnyi' [µg/mL]

Kruskal-Wallis test

Data	survival%
Factor	conc.
Filter	time="24 h"

Sample size	27

Descriptive statistics

Factor	n	Minimum	25th percentile	Median	75th percentile	Maximum
2.5	3	96.1430	96.443	97.343	98.742	99.209
10	3	99.1378	99.416	100.249	101.699	102.182
25	3	94.2434	95.159	97.906	102.273	103.728
50	3	94.9732	95.923	98.774	99.068	99.166
100	3	93.5413	94.102	95.786	100.718	102.363
200	3	89.0793	89.989	92.719	97.271	98.788
300	3	84.5038	85.512	88.537	88.638	88.671
500	3	71.6528	73.141	77.607	78.021	78.159
750	3	62.0671	64.177	70.508	70.969	71.122

Kruskal-Wallis test

Test statistic	20.5079
Corrected for ties	20.5079
Degrees of Freedom (DF)	8
Significance level	P = 0.008576

Post-hoc analysis (Conover)

Factor	n	Average Rank	Different (P<0.05) from factor nr
(1) 2.5	3	18.67	(7)(8)(9)
(2) 10	3	23.33	(6)(7)(8)(9)
(3) 25	3	19.33	(7)(8)(9)
(4) 50	3	18.33	(7)(8)(9)
(5) 100	3	17.67	(7)(8)(9)
(6) 200	3	13.67	(2)(8)(9)
(7) 300	3	8.00	(1)(2)(3)(4)(5)
(8) 500	3	5.00	(1)(2)(3)(4)(5)(6)
(9) 750	3	2.00	(1)(2)(3)(4)(5)(6)

Jonckheere-Terpstra trend test

Test statistic	47.000
Standard Error	23.812
z statistic	-4.830
P-value (two-sided)	<0.00001

Data: relative cell viability [%] for Mewo cells at 48h
Factor: concentration of C. mas 'Yantarnyi' [µg/mL]

Kruskal-Wallis test

Data	survival%
Factor	conc.
Filter	time="48 h"

Sample size	27

Descriptive statistics

Factor	n	Minimum	25th percentile	Median	75th percentile	Maximum
2.5	3	98.7241	98.982	99.754	103.773	105.113
10	3	101.3679	101.383	101.427	101.604	101.663
25	3	95.5368	96.516	99.453	106.160	108.395
50	3	97.4778	98.362	101.014	102.326	102.764
100	3	94.7460	95.193	96.533	102.329	104.260
200	3	83.6100	84.375	86.671	88.704	89.382
300	3	67.1323	68.104	71.019	74.438	75.578
500	3	50.9586	51.146	51.709	56.313	57.847
750	3	36.4771	36.578	36.881	43.338	45.491

Kruskal-Wallis test

Test statistic	21.9048
Corrected for ties	21.9048
Degrees of Freedom (DF)	8
Significance level	P = 0.005095

Post-hoc analysis (Conover)

Factor	n	Average Rank	Different (P<0.05) from factor nr
(1) 2.5	3	20.67	(6)(7)(8)(9)
(2) 10	3	22.00	(6)(7)(8)(9)
(3) 25	3	19.67	(6)(7)(8)(9)
(4) 50	3	20.00	(6)(7)(8)(9)
(5) 100	3	17.67	(6)(7)(8)(9)
(6) 200	3	11.00	(1)(2)(3)(4)(5)(9)
(7) 300	3	8.00	(1)(2)(3)(4)(5)
(8) 500	3	5.00	(1)(2)(3)(4)(5)
(9) 750	3	2.00	(1)(2)(3)(4)(5)(6)

Jonckheere-Terpstra trend test

Test statistic	36.000
Standard Error	23.812
z statistic	-5.292
P-value (two-sided)	<0.00001

Data: relative cell viability [%] for Mewo cells at 72h
Factor: concentration of C. mas 'Yantarnyi' [µg/mL]

Kruskal-Wallis test

Data	survival%
Factor	conc.
Filter	time="48 h"

Sample size	27

Descriptive statistics

Factor	n	Minimum	25th percentile	Median	75th percentile	Maximum
2.5	3	98.7241	98.982	99.754	103.773	105.113
10	3	101.3679	101.383	101.427	101.604	101.663
25	3	95.5368	96.516	99.453	106.160	108.395
50	3	97.4778	98.362	101.014	102.326	102.764
100	3	94.7460	95.193	96.533	102.329	104.260
200	3	83.6100	84.375	86.671	88.704	89.382
300	3	67.1323	68.104	71.019	74.438	75.578
500	3	50.9586	51.146	51.709	56.313	57.847
750	3	36.4771	36.578	36.881	43.338	45.491

Kruskal-Wallis test

Test statistic	21.9048
Corrected for ties	21.9048
Degrees of Freedom (DF)	8
Significance level	P = 0.005095

Post-hoc analysis (Conover)

Factor	n	Average Rank	Different (P<0.05) from factor nr
(1) 2.5	3	20.67	(6)(7)(8)(9)
(2) 10	3	22.00	(6)(7)(8)(9)
(3) 25	3	19.67	(6)(7)(8)(9)
(4) 50	3	20.00	(6)(7)(8)(9)
(5) 100	3	17.67	(6)(7)(8)(9)
(6) 200	3	11.00	(1)(2)(3)(4)(5)(9)
(7) 300	3	8.00	(1)(2)(3)(4)(5)
(8) 500	3	5.00	(1)(2)(3)(4)(5)
(9) 750	3	2.00	(1)(2)(3)(4)(5)(6)

Jonckheere-Terpstra trend test

Test statistic	36.000
Standard Error	23.812
z statistic	-5.292
P-value (two-sided)	<0.00001
