# Supplementary material for: Time-Resolved Metabolomics Reveals Distinct, Cell- and Variety-Dependent Profiles of Prostanoids in Melanoma Cells Exposed to Fruit Extracts from Cornus mas and C. officinalis: A Pilot Study
Source: Int J Mol Sci. 2026 Aug 3;27(15):6982. doi: 10.3390/ijms27156982 (PMC13467092; doi:10.3390/ijms27156982)
Supplement: Supplementary file 1 [file ijms-27-06982-s001.zip › Table S1.pdf]

Lewandowski et al.

Table S1. Median metabolite concentrations in untreated cells depending on their type and culture duration

Metabolite	Line	Duration	*N*	25th percentile	Median	75th percentile
PGE2	A375	24h	9	3.1	9.0	20.3
PGE2	A375	48h	9	14.7	24.7	39.7
PGE2	A375	72h	9	62.0	67.5	392.5
PGE2	MeWo	24h	9	192.8	210.7	287.2
PGE2	MeWo	48h	9	317.5	480.6	609.4
PGE2	MeWo	72h	9	327.9	415.5	523.4
PGF2a	A375	24h	9	5.9	10.1	30.4
PGF2a	A375	48h	9	15.5	22.7	34.2
PGF2a	A375	72h	9	38.1	97.1	203.2
PGF2a	MeWo	24h	9	342.1	452.0	616.8
PGF2a	MeWo	48h	9	491.0	703.2	925.7
PGF2a	MeWo	72h	9	416.8	615.8	803.9
PGJ2	A375	24h	9	60.5	156.0	414.8
PGJ2	A375	48h	9	120.6	447.0	826.9
PGJ2	A375	72h	9	108.0	252.7	519.2
PGJ2	MeWo	24h	9	156.7	223.7	269.0
PGJ2	MeWo	48h	9	133.9	177.5	243.4
PGJ2	MeWo	72h	9	171.4	251.6	316.2
PGD2	A375	24h	9	0.13	1.35	2.77
PGD2	A375	48h	9	0.00	1.02	1.37
PGD2	A375	72h	9	1.34	3.44	6.25
PGD2	MeWo	24h	9	19.06	22.82	38.01
PGD2	MeWo	48h	9	27.71	34.42	43.46
PGD2	MeWo	72h	9	23.85	31.91	44.84
PGE1	A375	24h	9	5.04	6.21	16.20
PGE1	A375	48h	9	5.12	7.05	18.09
PGE1	A375	72h	9	6.26	7.17	18.65
PGE1	MeWo	24h	9	5.82	6.01	6.88
PGE1	MeWo	48h	9	5.10	5.60	6.08
PGE1	MeWo	72h	9	5.67	6.58	6.93
PGF1a	A375	24h	9	0.20	0.54	0.64
PGF1a	A375	48h	9	0.13	0.25	1.23
PGF1a	A375	72h	9	1.37	1.78	3.22
PGF1a	MeWo	24h	9	0.40	0.78	1.09
PGF1a	MeWo	48h	9	0.34	0.66	1.93
PGF1a	MeWo	72h	9	0.76	0.83	1.82
TXB2	A375	24h	9	2.94	4.20	12.97
TXB2	A375	48h	9	5.45	11.96	24.30
TXB2	A375	72h	9	16.27	37.49	76.79
TXB2	MeWo	24h	9	7.50	8.61	9.63
TXB2	MeWo	48h	9	8.04	13.24	16.63
TXB2	MeWo	72h	9	9.99	10.73	15.27
LTB4	A375	24h	9	41.7	70.7	150.7
LTB4	A375	48h	9	70.5	141.8	232.1
LTB4	A375	72h	9	101.5	148.8	213.9
LTB4	MeWo	24h	9	62.9	74.1	89.3
LTB4	MeWo	48h	9	59.5	67.2	101.4
LTB4	MeWo	72h	9	60.1	78.8	92.9

N, numer of biological replicates.
