# Supplementary material for: Time-Resolved Metabolomics Reveals Distinct, Cell- and Variety-Dependent Profiles of Prostanoids in Melanoma Cells Exposed to Fruit Extracts from Cornus mas and C. officinalis: A Pilot Study
Source: Int J Mol Sci. 2026 Aug 3;27(15):6982. doi: 10.3390/ijms27156982 (PMC13467092; doi:10.3390/ijms27156982)
Supplement: Supplementary file 1 [file ijms-27-06982-s001.zip › readme.pdf]

README – Supplementary Materials

This supplementary file contains the complete statistical outputs for eight independent models, each corresponding to one prostaglandin. The analysis focuses on how the concentration of these metabolites changes over time in melanoma cell cultures exposed to different *Cornus mas* extracts at varying concentrations.

The models were built using a distributionally flexible framework that simultaneously estimates the mean level of the metabolite (mu), the variability (sigma), and the asymmetry of its distribution (nu). This approach allows the temporal trajectory of each metabolite to be modeled directly, while accounting for potential non-constant variance and non-normality in the data, which are common in biological systems.

The primary parameter of interest is timepoint_scaled, which represents the scaled time of observation. It captures how the concentration of the metabolite changes over time, independently of other experimental factors. The models also include baseline control terms for the melanoma cell line (celltype, either A375 or MeWo), the type of *Cornus mas* extract added to the culture (cornustype, including *Uholok* = black fruit, *Yantarnyi* = yellow fruit, and *Cornus officinalis*), the scaled concentration of the extract (cornusconc_scaled), and the replicate identifier (replication) to adjust for technical variation across experimental series.

The outputs presented here are from the final parsimonious models, where interaction terms that did not meaningfully contribute to the description of temporal dynamics were removed. This ensures stable estimation and biological interpretability.

Each model output includes three sections:

1. The full set of model coefficients for mu, sigma, and nu, directly as generated in R.
2. Diagnostic plots for residual evaluation, demonstrating the adequacy of the fitted model.
3. Simple effects, which are model-based predictions of how metabolite levels evolve over time under specific experimental scenarios.

Simple effects are not independent statistical tests and should not be interpreted using thresholds such as p-values. They are descriptive summaries of the model's predictions, intended to help understand the approximate magnitude and direction of temporal changes under specified conditions (e.g., at a given extract concentration or for a specific cell line). Their role is to support interpretation rather than to serve as confirmatory evidence of statistical significance.

In this study, changes in metabolite concentrations over time are modeled while controlling for baseline differences due to cell line identity, type and concentration of *Cornus mas* extract, and technical replicate effects. This allows us to isolate the temporal behavior of

each metabolite and generate mechanistic insights into the dynamic metabolic response of melanoma cells under experimental perturbation.

The structure of this file is as follows: each sheet corresponds to one metabolite, labeled Model 1 through Model 8. Within each sheet, results are organized in the order described above.
